# A synthetic protein-level neural network in mammalian cells

**DOI:** 10.1126/science.add8468

**Published:** 2024-12-12

**Authors:** Zibo Chen, James M. Linton, Shiyu Xia, Xinwen Fan, Dingchen Yu, Jinglin Wang, Ronghui Zhu, Michael B. Elowitz

**Affiliations:** 1 School of Life Sciences, Westlake University, Westlake Laboratory of Life Sciences and Biomedicine, Westlake Institute for Advanced Study, Hangzhou, Zhejiang, China; 2 Division of Biology and Biological Engineering, California Institute of Technology, Pasadena, CA 91125, USA; 3 Howard Hughes Medical Institute, California Institute of Technology, Pasadena, CA 91125, USA

## Abstract

Artificial neural networks provide a powerful paradigm for non-biological information processing. To understand whether similar principles could enable computation within living cells, we combined de novo designed protein heterodimers and engineered viral proteases to implement a synthetic protein circuit that performs winner-take-all neural network classification. This “perceptein” circuit combines weighted input summation through reversible binding interactions with self-activation and mutual inhibition through irreversible proteolytic cleavage. These interactions collectively generate a large repertoire of distinct protein species stemming from up to 8 co-expressed starting protein species. The complete system achieves multi-output signal classification with tunable decision boundaries in mammalian cells, and can be used to conditionally control cell death. These results demonstrate how engineered protein-based networks can enable programmable signal classification in living cells.

## One-Sentence Summary

A synthetic protein circuit that performs winner-take-all neural network computation in mammalian cells

## Introduction

Cells are classification machines. They use circuits of interacting genes and proteins to make qualitatively distinct decisions in response to the levels or dynamics of multiple input signals. For example, the p53 tumor suppressor pathway classifies the types and levels of stress the cell encounters, and induces senescence or cell death (1). During development, cells in the neural tube take on specific progenitor fates by classifying the amplitude of bone morphogenetic protein (BMP) and Hedgehog signaling (2). In the immune system, the classification of multiple cytokine inputs can control the fate of a T cell (3). The ability to program synthetic signal classification systems could facilitate engineered gene and cell therapies by allowing cells to distinguish diseased and normal cell states (4). A major goal of synthetic biology has been to design classification circuits that could control cellular outputs in response to different types of input signals (5).

**Figure 1.**
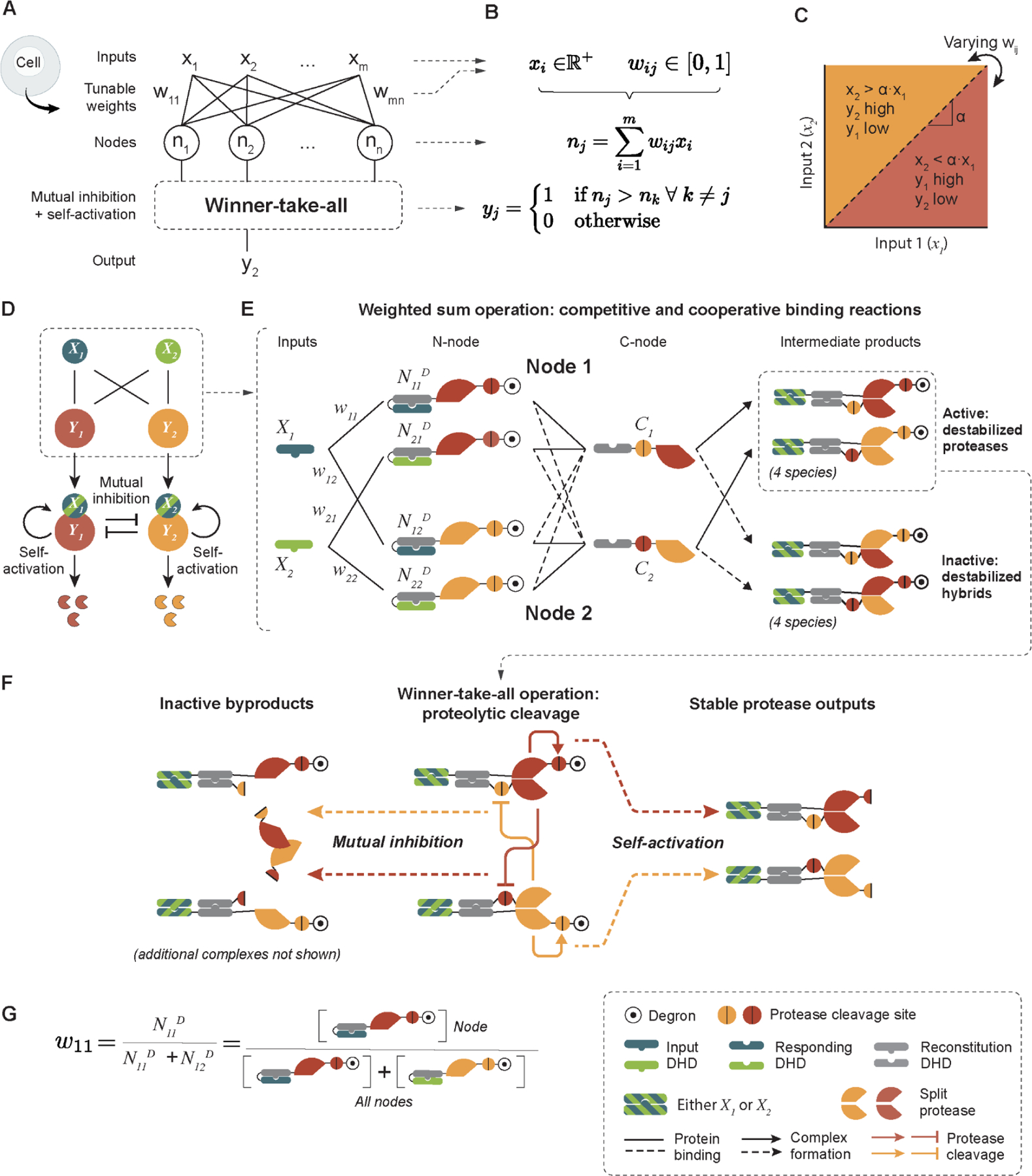
Winner-take-all neural network computation can be implemented using engineered proteins. (A) A winner-take-all neural network, operating inside cells, would use a set of interacting proteins (N_j_) to activate exactly one of its outputs (colored proteases, y_j_) depending on the relative values of its inputs (X_i_). (B) Formal description of the system in (A). The network consists of m inputs (X_i_), each taking positive real values. The inputs interact with the n nodes (N_j_) with weights w_ij_ (1 ≤ i ≤ m, 1 ≤ j ≤ n, 0 ≤ w_ij_ ≤ 1) connecting input X_i_ to node N_j_. Each node performs weighted sum operations to integrate the input signals it receives, and winner-take-all is achieved through self-activation and mutual inhibition. The output y_j_ from a node N_j_ is active only if its weighted sum is greater than that from any other node. (C) The decision boundary, α, for a 2-input, 2-node network can be tuned by varying the weights w_ij_. (D) In a 2-input, 2-node circuit, each input protein (X_1_, X_2_) activates either node protein (Y_1_, Y_2_) by forming input-node complexes. Such complexes then undergo self-activation and mutual inhibition to perform the winner-take-all computation. The final state of the system is defined by the abundance of the active node. (E) The weighted sum operation is carried out through competitive and cooperative binding. The two inputs are de novo designed orthogonal DHDs (X_1_ and X_2_). Each node consists of two groups of proteins: the N-nodes, where the cognate binding partners of X_1_ and X_2_ are caged by a genetically fused DHD caging domain, and further linked to the N-terminal half of a protease, its cleavage site, and a DHFR degron; the C-nodes, made from the cognate binding partner of the DHD caging domain in primary half-nodes, fused to the cleavage sequence of the other protease, and the C-terminal half of a protease. The inputs, N-node, and C-node bind cooperatively, such that neither of the two proteins can bind with high affinity without the third protein. They also interact competitively, such that the N-nodes compete to bind to the input protein, and the C-nodes. Two types of reconstituted proteases result from these 3-way binding events: the active but destabilized proteases where the two protease halves reconstitute a functional protease, or the inactive and destabilized hybrids where the two protease halves do not match. The blue and green stripes indicate that the DHD domains can be either blue (X_1_) or green (X_2_). (F) Winner-take-all operation is achieved through two types of reactions: mutual inhibition, where each protease can inactivate the opposite protease type by cleaving its C-terminal half proteases off the C-nodes; self-activation, where the intermediate DHD-protease complexes cleave off DHFR degrons from their N-nodes, converting them to stable proteases, which can in turn activate and inhibit other protease complexes. (G) The weights connecting inputs to nodes are set based on the abundance of each primary half-node complex. For example, w_11_, the weight that connects input X_1_ to Node 1, is defined as the concentration of the N_11_ N-node divided by the sum of the concentrations of all N-nodes that can potentially bind to X_1_, in this case N_11_ + N_12_.

One powerful classification architecture is the winner-take-all neural network (6). In these systems, an output node is ON if and only if the weighted sum of its inputs exceeds that of all other nodes in the output layer (Fig. 1, A and B). This architecture offers a compact mechanism for signal classification, requiring only a single layer neural network; ensures that outputs are all-or-none (Fig. 1B); and allows tuning of the decision boundary—the line separating points in input space that generate different outputs (Fig. 1C).

Theoretical work has suggested specific schemes for engineering biochemical neural computation systems (7–13). Experimentally, efforts to build synthetic classification systems have resulted in DNA-based (14, 15) or enzymatic (16) classifiers in test tubes, and miRNA-based (17, 18) or genetic (19–21) classifiers in cells. A protein-level classifier would offer several advantages: it could be expressed transiently in cells and interface directly with endogenous inputs and outputs, circumventing transcriptional delays. It should also work in diverse cell types without relying on endogenous transcriptional regulation. More generally, creating a protein-based neural network would demonstrate the feasibility of constructing complex computational systems out of interacting proteins (22).

Two advances in synthetic protein biology facilitate the design of protein-based neural networks. First, sets of modular binding domains, including *de novo* designed heterodimers (DHDs), allow programmable control of protein interactions (23–26). Second, those interactions can in turn be used to reconstitute split viral proteases, and thereby control their activity (27–30). Leveraging these advances, we designed protein-based winner-take-all circuits using DHDs and split proteases, and showed that they can classify the relative abundance of input proteins in living mammalian cells.

## Results

### System design

Three key circuit features enable winner-take-all dynamics (Fig. 1D): First, weighted summation of inputs allows each node in the neural network to respond to each input molecule with a tunable strength. Second, mutual inhibition between output nodes eliminates less abundant species. Third, self-activation of each output node allows surviving species to amplify and sustain their own activity (31).

We designed a set of chimeric proteins that collectively provide these features. As inputs, we constructed a set of “input DHD” monomers (Fig. 1E, blue and green bulged shapes). These inputs then bind to and activate proteins termed N-nodes, which contain N-terminal protease halves. Once activated, the N-nodes bind to C-nodes that contain C-terminal protease halves. The N- and C-node proteins include other DHD domains, protease cleavage sites, and—for N-nodes—the dihydrofolate reductase (DHFR) degron (Fig. 1E, N-node and C-node). We conceptually label N- and C-nodes containing the same type of viral protease together as a “node.” Output levels from each node are represented by the concentrations, and hence overall activities, of each reconstituted protease species (Fig. 1F, stable protease outputs; Fig. S1).

Each input protein can activate each node. In the absence of inputs, the responding DHDs (Fig. 1E, blue and green domains with notches) and reconstitution DHDs (Fig. 1E, gray bulged shapes) in each N-node subunit self-cage through an intrinsically weak intramolecular binding interaction. However, when input DHDs are present, they can outcompete this interaction and effectively uncage the reconstitution DHD domains (Fig. S2A) (32). This frees the reconstitution DHDs on the N-node subunits to pair with cognate domains on the C-nodes (Fig. 1E, reconstituted proteases; Fig. S2A), resulting in either a functional protease (Fig. 1E, top right) or a non-functional hybrid of mismatched protease halves (Fig. 1E, bottom right; blue and green stripes indicate species that could contain either input DHD specificity), with the latter contributing to mutual inhibition. All node proteins were designed to share the same reconstitution domain in order to ensure similar protease reconstitution kinetics. Altogether, this scheme allows each input protein to activate each node.

This design also enables weighted summation of inputs. Each input species can simultaneously interact with multiple N-node subunits through corresponding DHD binding partners. Mass action partitions each input across the N-nodes in proportion to their relative abundances (Fig. 1E, left). The effective weights can thus be controlled by the relative abundance of expressed N-node proteins. For example, the weight connecting input 1 to node 1 can be encoded as the proportion of node 1 N-node proteins to all N-node proteins that can bind to input 1 (Fig. 1G, (33)). Input summation then occurs naturally because the total amount of reconstituted protease of a given type includes contributions from all inputs.

Finally, this circuit design uses protein degradation and protease cleavage to achieve self-activation and mutual inhibition, respectively. To realize self-activation, we fused protease-cleavable DHFR degron domains to the N-node proteins, resulting in their rapid degradation in the absence of inputs. Once reconstituted, however, functional proteases stabilize their own concentrations by cleaving N-node subunits of the same protease type at engineered cleavage sites, and thereby removing the DHFR degrons (Fig. 1F, right). Further, functional reconstituted proteases of each type can also inactivate functional proteases of other types by cleaving off the reconstitution DHDs from the C-nodes (Fig. 1F, left).

Taken together, these components thus implement weighted summation, mutual inhibition, and self-activation, and therefore could support winner-take-all classification. We term these systems perceptein networks, in loose analogy with the Perceptron, a foundational artificial neural network architecture (34).

### Modeling and simulation

To represent protein-protein interactions, we introduce a simplified notation, based on the assumption that DHD binding occurs only between cognate domains (Fig. 1E, Table S2) (33). Briefly, we denote input DHDs by X_i_, and reconstituted protease outputs by Y_k_, where i and k respectively index the input and output species. We use C_k_ to denote the C-node with half-protease species k. Finally, we write N_ij_^D^ and N_ij_ to represent the N-node proteins that interact with input i and contain half-protease j, either with or without the degron, respectively. In the simple case of two inputs and two outputs, there are a total of 8 starting protein species: two X_i_, four N_ij_^D^, and two C_k_, with all subscripts ranging from 1 to 2. X_i_ binds to N_ij_^D^ or N_ij_, and the resulting dimer can bind to C_k_ to reconstitute either functional full proteases (j=k) or nonfunctional hybrids (j≠k).

**Figure 2.**
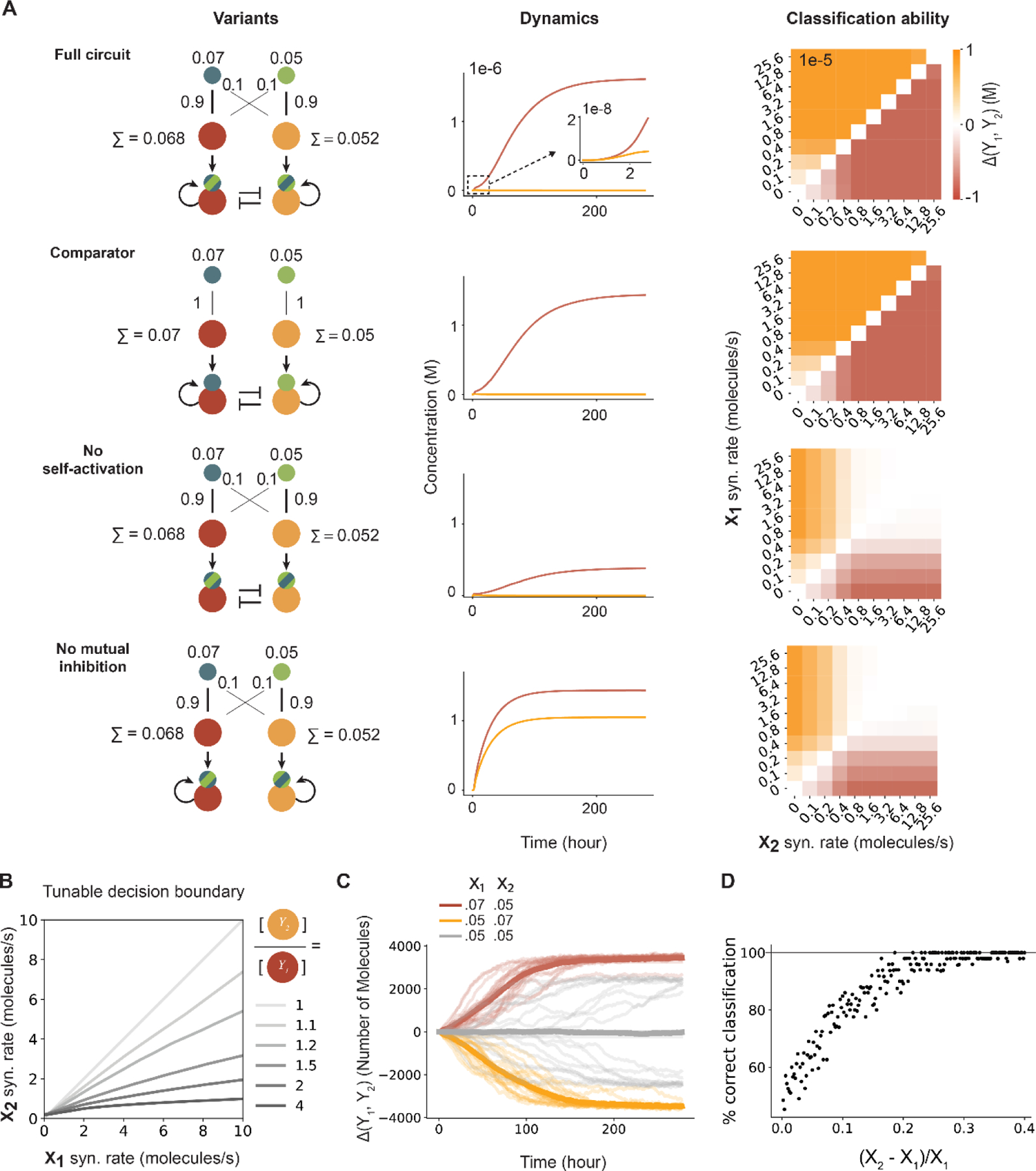
Simulated two-input circuits perform winner-take-all classification. (A) Simulations of circuit variants (left) reveal circuit dynamics (middle) and classification ability (right). Input values, weights, and weighted sums (denoted Σ) are indicated on the circuit diagrams, with larger weights represented by thicker lines. Each cell in the heatmap represents the difference between active Y_1_ and Y_2_ proteases at steady state. Both the full circuit and the comparator are able to classify across the full range of input levels, while circuits lacking self-activation or mutual inhibition only classify within a limited input range. (B) The decision boundary (gray lines) of the comparator circuit can be tuned by varying the relative levels of the two node proteins, N_11_^D^ and N_22_^D^. (C) Stochastic simulations of the comparator. Twenty simulations were performed for each condition (light traces), and their average traces are plotted in dark lines. Colors indicate the input levels (legend). See supplementary materials for simulation methods. (D) Percentage of 50 equivalent simulations that correctly classify inputs as a function of the concentration difference between the two inputs. Input X_1_ is fixed at 0.05 molecules/s. Even with stochasticity, input differences of at least 20% classify correctly ~95% of the time.

To understand the expected behavior of this system in physiologically relevant parameter regimes, we simulated its response to a matrix of input values (X_i_ concentrations) (33). Due to protein cleavage and alternative complex formation, the perceptein circuit produces an enormous number of distinct molecular species (Fig. S1). For example, in its simplest implementation, involving just two inputs and two outputs (Fig. 2A, first row), the system generates, from 8 initial protein species, 158 unique proteins and protein complexes that participate in 310 distinct chemical reactions (33).

To represent the cooperative binding step in these equations, we assumed that inputs, X_i_, bind cooperatively to nodes, N_ij_^D^ and C_k_ to form trimeric complexes that reconstitute either functional proteases or nonfunctional hybrids with mismatched protease halves (33, 35). More specifically, we divided trimer formation into two steps. First, X_i_ binds to N_ij_^D^ to form an unstable dimer with large off rates (Table S1), exposing the binding site for C_k_. Second, this dimer binds to C_k_ to form stable trimeric complexes that have slower off rates than those of complexes lacking N_ij_^D^ (32). We also allow each reconstituted protease to cleave its cognate target sites on other protein components. All the while, all protein building blocks are continuously synthesized at constant rates and degraded at a basal or elevated rate depending on whether they contain a degron. Finally, we estimated physiologically reasonable values for protein synthesis and degradation rates, protease catalytic rates, and other biochemical parameters using references in the BioNumbers (36) database (Table S1).

The simulated circuit successfully exhibited winner-take-all classification behavior (Fig. 2A, first row). When X_1_ exceeded X_2_, the Y_1_ protease activation accumulated to its maximum possible level over timescales of several days (~100h). However, the decision was made much earlier, as large fold differences between Y_1_ and Y_2_ were apparent within 3 hours (Fig. 2A, first row inset). Simulations further revealed that winner-take-all classification functioned across a broad range of absolute concentrations for X_1_ and X_2_, and remained accurate even for differences as small as 10% between the two ligand concentrations (Fig. S2B).

To understand which features of the circuit were necessary or sufficient for classification, we simulated circuit variants lacking cross-activation by inputs (Fig. 2A, second row), self-activation (Fig. 2A, third row), or mutual inhibition (Fig. 2A, fourth row). Removing cross interactions (i.e., removing N_12_^D^ and N_21_^D^) produced a simplified “comparator” circuit design (Fig. 2A, second row) that still allowed winner-take-all classification. Removing self-activation retained the all-or-none behavior at lower input levels, but reduced the output dynamic range (Fig. 2A, third row) and, at higher input levels, eliminated classification ability altogether. By contrast, removing mutual repression accelerated the response of the circuit but eliminated the all-or-none output behavior, and classification ability at higher input levels (Fig. 2A, bottom row). These results indicate that self-activation and mutual inhibition are indispensable for winner-take-all computation in this architecture.

Sensitivity analysis revealed that protease catalytic rates and protein degradation rates have strong effects on classification accuracy (Fig. S2, C and D, (33, 35)), while the decision boundary for classification can be tuned by varying weights, represented here as component concentrations. For example, in simulations, varying the relative abundances of N_11_^D^ and N_22_^D^ allowed the construction of biased comparators, where Y_2_ is ON only whe‧n X_2_ > X_1_ᐧα, where α depends on the relative levels of N_11_^D^ and N_22_^D^ (Fig. 2B).

Stochastic fluctuations, or ‘noise,’ can impact circuit behaviors in cells (37). Stochastic simulations revealed that both the full network and the comparator circuits could tolerate physiologically reasonable levels of noise (Fig. 2C, Fig. S2 E-G). For example, with a difference in input values of 40% (e.g., X_1_=0.05, X_2_=0.07), no “reversal” events were observed, as shown by individual traces (red and orange lines, Fig. 2C). On the other hand, when starting from equal inputs (X_1_=X_2_=0.05), individual trajectories converged over a slower timescale to either of the two output states, in a bistable fashion (Fig. 2C). Finally, differences in input concentrations of only 20% were sufficient for accurate classification more than 95% of the time (Fig. 2D). Taken together, these simulations suggested that the perceptein architecture could function across a broad range of physiologically reasonable parameter values.

### Experimental validation

**Figure 3.**
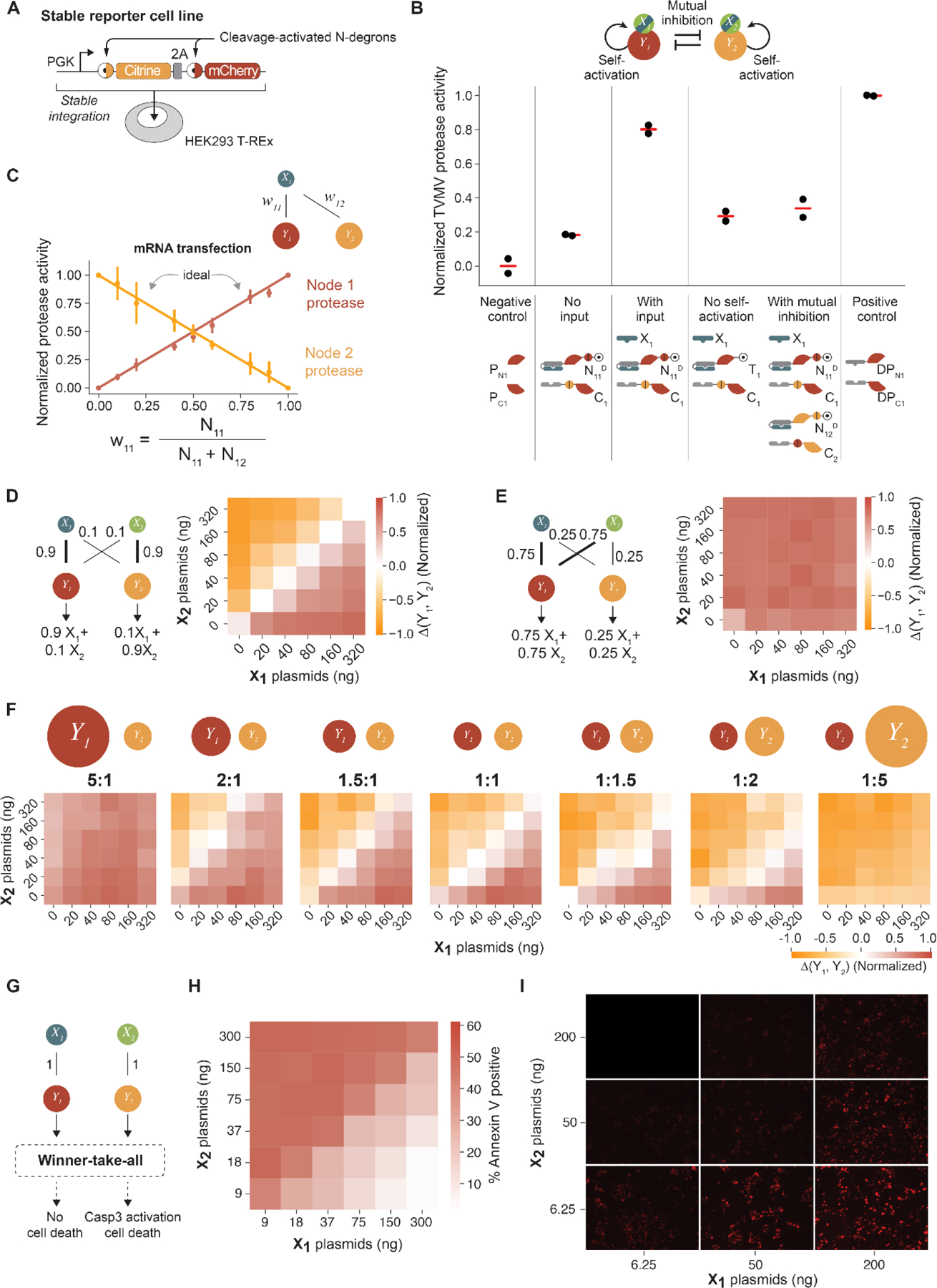
The winner-take-all neural network circuit classifies inputs in mammalian cells. Unless otherwise noted, transfections were conducted using DNA. (A) The stable reporter cell line constitutively co-expresses Citrine and mCherry fluorescent proteins that can be cleaved at the N-terminus by TEVP and TVMVP, respectively, to reveal N-terminal degrons that destabilize the fluorescent proteins. PGK, 3-phosphoglycerate kinase promoter. (B) Engineered protease can respond to inputs, self-activate, and mutually inhibit. Normalized protease activities under different experimental setups indicate expected functions. (C) Testing the weight multiplication module by fixing input X_1_ and varying nodes N_11_^D^ and N_12_^D^. Ideal behaviors are shown in solid lines, experimental data points from mRNA transfections are mean ± s.d from three biological repeats. (D) A fully connected 2-input, 2-node circuit that compares relative input levels (left). (E) A fully connected 2-input, 2-node circuit that, by construction, should always result in Y_1_ being the winner. (F) The decision boundaries of a two-input comparator can be tuned by varying the ratios of Y_1_ to Y_2_ protein concentrations. Data in D-F are averages of two biological replicates. (G) A cell death circuit is placed downstream of the perceptein classifier, where activation of Node 2 results in cell death. (H) HEK293 cells were transfected with the perceptein-apoptosis circuit (two inputs, two nodes, and TEVP-activatable caspase-3) and an mCherry co-transfection marker. The percentage of apoptotic (Annexin-positive) cells were calculated using flow cytometry, after gating on transfected cells based on an mCherry co-transfection marker. Data points are averages of four biological replicates. (I) Red fluorescence images of cells treated with the perceptein-apoptosis circuit along with the mCherry co-transfection marker. Dead cells show reduced or no expression of mCherry.

To determine whether perceptein circuits could operate in mammalian cells, we experimentally constructed the set of 6 perceptein protein components and 2 input proteins necessary to implement the full two-input, two-output circuit (Fig. S3). We chose the well-characterized split tobacco etch virus protease (TEVP) and tobacco vein mottling virus protease (TVMVP) (27) as the two orthogonal proteases, and genetically fused their split halves to DHD domains, protease cleavage sequences, and degradation domains for controlled reconstitution of full proteases (Fig. S3, Table S2). To facilitate analysis of various component combinations and expression levels by co-transfection, each protein was encoded on a distinct plasmid. To measure the output of the circuit, we engineered a stable human embryonic kidney (HEK) 293 reporter cell line (HEK1012, (35)) containing a multi-cistronic construct co-expressing two fluorescent proteins—Citrine and mCherry—each tagged with a cleavage-activated N-degron for either of the two input proteases (27) (Fig. 3A, S4A). We verified that each protease variant exclusively reduced fluorescence from its target reporter (Fig. S4B). Together, these constructs and the reporter cell line permitted rapid, iterative testing of circuit designs using flow cytometry.

We experimentally validated each module of the winner-take-all neural network (Fig. 1D). We tested whether inputs could trigger reconstitution of corresponding protease activities (Fig. 1E). Co-transfecting input X_1_ with cognate N- and C-node proteins inactivated the corresponding fluorescent protein reporter, consistent with reconstitution of the corresponding protease (Fig. 3B, S4C, S4D). Input-triggered protease activities were comparable to those of positive controls, consisting of split protease halves fused to heterodimerizing domains. In the absence of input, reporter levels were similar to those in a negative control consisting of split protease halves lacking DHD domains. These results suggest that inputs reconstituted cognate protease activities, as expected.

Next, we evaluated the weight multiplication step, which should ideally distribute input proteins based on the relative abundance of N-node proteins (Fig. 1G). We transfected cells with various ratios of N_11_ and N_12_ mRNA, while keeping X_1_ constant. We separately verified that these transfected mRNAs were stable in HEK293 cells, with decay rates of 13.8 ± 2.3 hours (Fig. S5A). The amount of activated (protease reconstituted) Y_1_ and Y_2_, which determines the fluorescence of Citrine and mCherry, should ideally be linearly dependent on the ratio of transfected N_11_ and N_12_ constructs. This behavior was largely confirmed by flow cytometry analysis (Fig. 3C).

Once activated, nodes should self-activate and mutually inhibit, as described above, by cleavage at appropriate target sites (Fig. 1F). To experimentally verify self-activation, we transfected HEK293 cells with plasmids encoding either N-node constructs with a protease cleavable degron, or negative control constructs lacking the cleavage site that were therefore unable to self-activate. Flow cytometry revealed that signals from the latter configuration remained close to background, as expected (Fig. 3B and S4E). Mutual inhibition interactions (Fig. 1F) also functioned as expected. Protease activities of the Y_1_ node were strongly repressed by an excess of the Y_2_ node components (N_12_^D^ and C_2_). Mutual inhibition worked similarly in the opposite direction when there was more Y_1_ than Y_2_ (Fig. S4E). These results indicate that each module of the full circuit can function individually.

To test the full circuit, we transfected reporter cells (Fig. 3A) with various concentrations of the inputs, fixed amounts of the N- and C- half-node proteins (in a multicistronic manner), and a blue fluorescent protein (BFP) co-transfection marker (Fig. S3), and read out reporter fluorescence. We set the concentrations of the N_11_^D^ and N_22_^D^ plasmids 9 times higher than the concentrations of the N_12_^D^ and N_ij_^D^ plasmids to put the circuit into a comparator regime (Fig. 3D, left). We normalized protease activities based on fluorescence, and plotted the differences in normalized protease activities in Y_1_ and Y_2_. As expected, the output was positive when X_1_ exceeded X_2_ and negative when X_2_ exceeded X_1_, with minimal response at equal input concentrations (Fig. 3D, right). The output became more binary with greater absolute difference between the two inputs, approaching the binary response observed in simulations. The experimental classification result resembles output from the softmax function, which is commonly used in artificial neural networks as the activation function in the output layer (Fig. S5B). Additionally, varying the relative levels of perceptein components resulted in a biased comparator where, in agreement with prediction (Fig. 3E, left), Node 1 was the winner regardless of the two input values (Fig. 3E, right). These results show that the full circuit can compare the relative concentrations of two inputs.

The fully connected neural network is useful in classification tasks where there are more inputs than the number of nodes. However, when the number of inputs equals the number of nodes, eliminating the cross interactions, X_1_-Y_2_ and X_2_-Y_1_, leads to a simpler comparator that still allows input classification with a tunable decision boundary (Fig. 2A, second row). To test this capability, we transfected cells with various relative amounts of the N_11_^D^ and N_22_^D^ plasmids, but omitted the N_12_^D^ and N_21_^D^ plasmids entirely. At a 1:1 ratio of N_11_^D^:N_22_^D^, the comparator achieved similar classification behavior to that observed with the full circuit (Fig. 3D), with dynamics that closely agreed with results from simulations (Fig. 2A, S4F).

The system was also robust to variation in the timing of input addition, achieving similar decision outcomes when the input plasmids were transfected 24 hours after transfecting the node plasmids, consistent with model predictions (Fig. S2H, S4G). Further, modulating the relative abundance of N_11_^D^ and N_22_^D^ shifted the decision boundary, allowing tuning (Fig. 2B, 3F). Analysis of transiently transfected cells at the bulk level did not show perfectly digital winner-take-all behavior (Fig. 3D, F), possibly due to effects of intrinsic noise observed in stochastic simulations (Fig. S5C). Nevertheless, these results support the ability of the circuit to experimentally enable tunable classification.

The protein nature of the perceptein circuit enables it to directly interface with endogenous pathways. To demonstrate this, we linked the perceptein output to a caged caspase-3 that is activated upon TEVP cleavage to trigger apoptosis (Fig. 3G) (38), changing the classification outcome from fluorescence to life-or-death. HEK293 cells transfected with the perceptein cell death circuit were able to correctly classify themselves into living or dead cells in response to various input combinations (Fig. 3H, 3I). These results demonstrate that the perceptein circuit can be used to control cellular processes.

### Scalable classification

**Figure 4.**
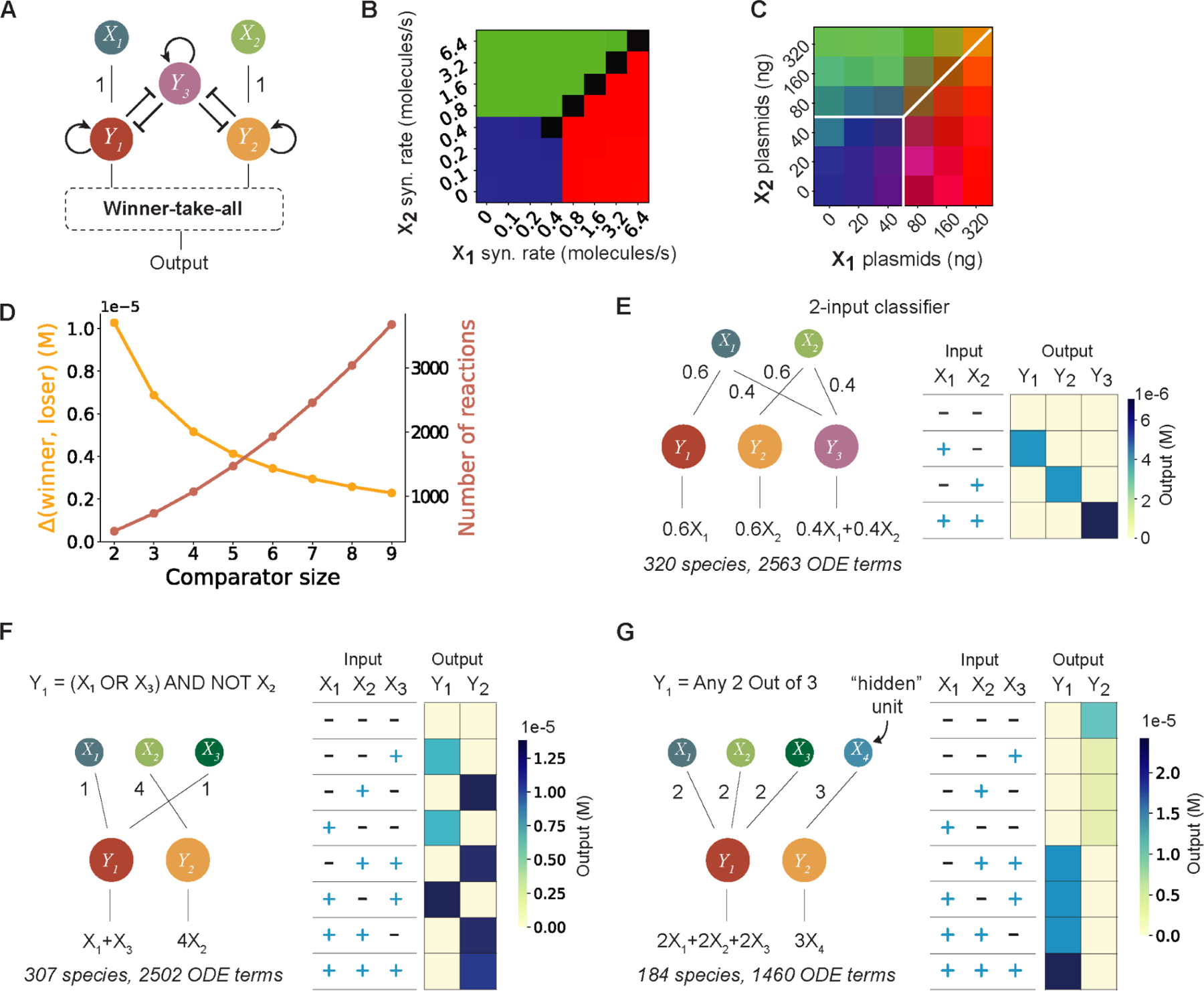
Scaling of the winner-take-all circuit. (A) A thresholded two-input comparator where the third node (Y_3_) participates in self-activation and mutual inhibition, but does not directly respond to inputs (X_1_ and X_2_). (B) Simulation results predict three distinct classification outcomes (red, green, and blue) depending on input levels, and a fourth unclassified state (black). (C) The thresholded two-input comparator was tested in a three-color reporter cell line transiently transfected with perceptein components. For each cell, normalized fluorescent levels from the Citrine, mCherry, and miRFP680 channels were converted to a three-element RGB vector that represents the color of the cell. Data points represent averages of three biological replicates. (D) The number of reactions in a comparator circuit increases roughly linearly with its size, as the circuit dynamic range decreases. (E) A 2-input classifier can generate distinct responses to all 4 input states. (F) A 3-input winner-take-all circuit performs the (X_1_ OR X_3_) AND NOT X_2_ calculation. Node 1 wins if the condition is met. (G) A 3-input winner-take-all circuit performs “Any 2 out of 3” logic. A fourth “hidden unit” input was added to set the threshold and make the circuit more compact. Node 1 wins if the condition is met.

A key feature of neural networks is their ability to scale computational power with network size. In simulations, incorporating an additional constitutively active node (i.e., one that does not respond to any input species) that mutually inhibits the two other nodes can serve as a thresholding module, giving rise to a third classification outcome in the two-input comparator (Fig. 4, A and B). To experimentally verify this prediction, we selected the Human Rhinovirus 3C Protease (HRV3CP) as the third node, and constructed a stable reporter cell line (HEK1013, (35)) constitutively expressing Citrine, mCherry and miRFP680 with N-end protease activatable degrons upon TEVP, TVMVP, and HRV3CP cleavage, respectively (Fig. S6 A-C). After transfecting plasmids encoding all protein components (Fig. S3), HEK293 cells could indeed classify input signals into three distinct categories, in close agreement with simulation results (Fig. 4C). These data reveal that the perceptein network can expand as orthogonal proteases are added, and suggests that further expansion might be possible as new proteases are discovered or engineered.

To explore how much further this protein-based neural network might scale, we simulated higher dimension comparators, each composed of *m* inputs and *m* nodes (Fig. S6D). For each value of *m*, we simulated the response to a matrix of input values. Larger systems retained classification ability, despite some loss of output dynamic range (Fig. S6E). Overall circuit complexity, measured by the number of chemical reactions, scaled approximately linearly with the size of the comparator, *m* (Fig. 4D).

Finally, simulations showed that the perceptein system can also perform more complex types of classification. For example, by adding a third node to the two-input classifier, one can obtain a winner-take-all response in which nodes 1 and 2 respond to X_1_ or X_2_ alone, while node 3 responds only to the presence of both (Fig. 4E). Conversely, with three inputs and two nodes one can, in a single layer system, compute composite functions such as (X_1_ OR X_3_) AND NOT X_2_ that require multiple layers of conventional Boolean logic gates (Fig. 4F). One can also augment the system with “hidden” units that establish thresholds for the true inputs in order to compute even more complex functions. For example, by adding a single hidden unit to the 3-input, 2-node system, one can obtain a circuit that computes an ANY 2 OUT OF 3 function, where node 1 is activated when at least two of the three inputs are present (Fig. 4G). Thus, the perceptein system can in principle be scaled and extended to solve a broader variety of classification problems.

## Discussion

Early synthetic biology focused on importing digital logic paradigms from computer science to biology (39, 40). However, natural biochemical networks often resemble neural networks in their dense webs of many-to-many interactions. These observations provoke the question of whether it is possible to build neural network-inspired biochemical circuits synthetically. Here, we demonstrate that *de novo* designed protein domains and engineered enzymes can be combined to implement a classic winner-take-all neural network classification network, and allow tunable control of decision boundaries in living cells.

In the perceptein design, a large number of different protein species and complexes (e.g., 158 for the 2-input, 2-output circuit) are produced in the operation of this system, but effectively “compressed” into a much smaller number (e.g., 8 for the 2-input, 2-output circuit) of starting protein species from which they are generated. This is reminiscent of the way some protein families produce huge diversities of protein products through alternative splicing, proteolytic cleavage of pro-proteins (41), or combinatorial assembly of alternative multimeric complexes (42).

While they demonstrate simple neural computation, the circuits described had limitations. Classification outcomes were not digital, possibly reflecting expression variability of transient transfection methods. Additionally, network size is currently limited by the number of reliable engineered proteases, as well as technical difficulties in expressing large numbers of proteins in a controlled way. Finally, extending the full circuit (or comparator) to more complex computations requires a multiplicative (or linear) increase in the number of components.

Implementing large-scale computational circuits in cells may require the ability to construct many enzyme variants with programmable specificities, improvements in engineering of large multi-gene expression systems (43, 44), and more efficient naturally inspired architectures (45). Nevertheless, we anticipate that as genome engineering techniques continue to advance (46), permitting creation of larger systems, neural circuit architectures will extend the computational capacities of living cells.

## Supplementary Material

1

## Data Availability

Raw data and code used for simulation can be downloaded from https://data.caltech.edu/records/ep884-g0v97. Plasmids and cell lines are available upon request.
