# Supplementary material for: A synthetic protein-level neural network in mammalian cells
Source: Science. Author manuscript; Available in PMC 2025 Jan 24. (PMC11758091; doi:10.1126/science.add8468)
Supplement: 1 [file NIHMS2043306-supplement-1.pdf]

Supplementary Materials for

A synthetic protein-level neural network in mammalian cells

Zibo Chen, James M. Linton, Shiyu Xia, Xinwen Fan, Dingchen Yu, Jinglin Wang, Ronghui Zhu, Michael B. Elowitz

Correspondence to: zibochen@westlake.edu.cn (Z.C.); melowitz@caltech.edu (M.B.E.)

This PDF file includes:

Materials and Methods
Supplementary Text
Figs. S1 to S6
Tables S1 to S3

Materials and Methods

Overview of experimental workflow

Our main experimental approach involves transiently co-transfecting mRNA or plasmids encoding perceptein components (listed in Table S3) into HEK293 and derived reporter cells, inducing expression of proteins by adding doxycycline, and performing flow cytometry assays 48 hours post transfection.

Construction of synthetic genes

Some constructs were generated using standard cloning procedures. The inserts were generated using PCR or gBlock synthesis (IDT), and were annealed by Gibson assembly with backbones that are linearized using restriction digestion. The remainder were designed by the authors and synthesized by Genscript. mRNAs were ordered from TriLink BioTechnologies. A list of all constructs used in this study is included in Table S2.

Tissue culture

The monoclonal reporter cell line HEK1012 was generated using the PiggyBac Transposon system (Systems Biosciences) in the Flp-In™ T-REx™ Human Embryonic Kidney 293 cell line (HEK293 T-REx, Thermo). The plasmid harboring a transposon with the 3-phosphoglycerate kinase (PGK) promoter driving the expression of Citrine and mCherry with N-end protease activatable degrons was co-transfected with a plasmid encoding the Super PiggyBac Transposase into HEK293 T-REx cells. 24 hours after transfection, cells were transferred into a 6-well plate and selected with 400 μg/ml Zeocin for 9 days (split into Zeocin media every 3 days). The resulting polyclonal cells were then diluted at ~ 1 cell/well into 96-well plates. After a week, wells with single clones showing positive Citrine and mCherry fluorescence were selected. The HEK1012 line was derived from one of the clones found to have medium Citrine and mCherry expression from flow cytometry measurement. The monoclonal reporter cell line HEK1013 was derived from HEK1012 by further integrating a cassette containing the CMV early enhancer/chicken beta actin (CAG) promoter driving the expression of miRFP680 with N-end protease activatable degrons. Cells were maintained in Eppendorf CellXpert cell culture incubators at 37°C with 5% CO_2. Cells were grown in media containing Dulbecco's Modified Eagle Medium (Gibco) supplemented with 10% Fetal Bovine Serum (Avantor), 1 mM sodium pyruvate (Gibco), 10 unit/ml penicillin (Gibco), 10 μg/ml streptomycin (Gibco), 2 mM L-glutamine (Gibco) and 0.1 mM MEM non-essential amino acids (Gibco).

Transient transfection of DNA into reporter cells

HEK1012 or HEK1013 reporter cells were seeded in 24-well plates at a density of $0.05\sim0.1*10^6$ cells per well and cultured for 24 hours. Transient transfection was performed the following day using Lipofectamine 2000 (Thermo Fisher) following the manufacturer's protocol. Doxycycline was then added to the growth media at 100 ng/mL to induce expression from the CMV-TO promoter. Cells were changed into fresh growth media, with 100 ng/mL doxycycline, 24 hours after transfection, and were analyzed by flow cytometry after another 24 hours.

Transient transfection of mRNA into reporter cells

HEK1012 reporter cells were seeded in 24-well plates at a density of $0.05*10^6$ cells per well and cultured for 24 hours. Transient transfection was performed the following day using the TransIT®-mRNA Transfection Kit (Mirus Bio) following the manufacturer's protocol. Cells were analyzed by flow cytometry after 24 hours.

<u>Flow cytometry</u>
Cells were trypsinized with 40 µL of 0.05% trypsin-EDTA (Gibco) for 1 minute at room temperature, and subsequently resuspended in 100 µL of Hanks' Balanced Salt Solution (HBSS) containing 2.5mg/ml bovine serum albumin (BSA), 1mM ethylenediaminetetraacetic acid (EDTA), and/or 4 units/ml DNase I (NEB). Cells were then filtered through a 40 µm cell strainer (Falcon™) or a 96-well plate cell strainer (Millipore) and analyzed by flow cytometry (CytoFLEX, Beckman Coulter or ZE5, Bio-Rad).

<u>Fluorescent signal quantification</u>
Flow cytometry data was processed using the Cytoflow python package (https://github.com/cytoflow/cytoflow). Events collected from flow cytometry experiments were first gated based on forward vs. side scatter to select for cells, followed by gating based on forward scatter area vs forward height, to select for single cells. Data were then gated on fluorescence of the blue fluorescent protein (BFP), emission 450/45, co-transfection marker between 98 and 99.5 percentiles. Median values were taken from Citrine, 525/40, mCherry, 610/20, or miRFP680, 720/60, output signals.

<u>Cell death assay</u>
HEK293 cells were transfected with TEVP-activatable caspase-3 in addition to perceptein circuits (Table S3) using Lipofectamine 2000 (Thermo Fisher). At 24 hours post transfection, supernatants containing dead cells were collected and pooled with remaining cells lifted using 0.05% trypsin-EDTA (Gibco). The cells were spun at 200 g for 5 min. Then, supernatants were carefully removed and pellets were resuspended in HBSS containing 2.5 mg/ml BSA and 2.5 mM calcium chloride. Pacific blue-conjugated Annexin V (Invitrogen) was added to the resuspended cells before analysis by flow cytometry (CytoFLEX, Beckman COulter).

<u>mRNA stability assay</u>
HEK293 cells were seeded in a 24-well plate at $0.15*10^6$ cells/well and cultured for 16 hours. They were then transfected with 200 ng of mTagBFP2-encoding mRNA using the TransIT®-mRNA Transfection Kit (Mirus Bio) following the manufacturer's protocol. Post 16 hours, cells were reseeded from each well into 3 new wells, yielding 18 wells in total.

After a further 8 hours, cells in three wells were lysed using 0.35 ml of RLT buffer (Qiagen) and stored at -80°C. This procedure was repeated for the remaining wells at 24, 48, 72, 96, and 120 hours. In addition, lysates from two untransfected control wells were prepared as negative controls for mTagBFP2 expression. Subsequent real time qPCR (RT-qPCR) confirmed that these samples produce cycle thresholds (Ct) not above noise.

RNA was extracted from all lysates using the RNeasy kit (Qiagen). RNA concentrations were determined with a Nanodrop 8-channel spectrophotometer (Thermofisher). cDNA was synthesized from 1 µg of each RNA sample using iScript (Bio-RAD). All procedures were done as per the manufacturer's recommended conditions.

For mTagBFP2 copy number quantification, IQ SYBR Green Supermaster mix (Bio-Rad) was employed using 1:40 of cDNA from samples. RT-qPCR was performed on a CFX96 (Bio-Rad) using the forward primer CTAGGGAGGTCGCAGTATCT and reverse primer CTATGTGGACTACAGACTGGAAAG. A standard curve was generated using three replicates each of known mTagBFP2 plasmid copies. The Cts from the standards informed mTagBFP2 copy number in test samples.

Calculation of protease activities

Because the reporter cell lines are constitutively expressing protease repressible fluorescent proteins, normalized protease activities were calculated using (Neg-Obs)/(Neg-Pos), where Neg is observed fluorescence when cells were transfected with the negative control plasmids (protease halves), Pos is observed fluorescence when cells were transfected with the positive control plasmids (protease halves fused to DHD domains), and Obs is observed fluorescence in each experiment.

In each 6*6 titration matrix experiment, protease activities were normalized using (Max-Obs)/(Max-Min), where Max is the fluorescence from the well where the protease activity is predicted to be the lowest based on our simulation, Min is the fluorescence from the well where the protease activity is predicted to be the highest based on our simulation, and Obs is observed fluorescence in each well. For the thresholded two-input comparator experiment in HEK1013 cells (Fig. 4C), normalized fluorescent levels from the Citrine, mCherry, and miRFP680 channels were converted to a three-element RGB vector with each value ranging from 0 to 255. Combining these three elements into a RGB vector gives rise to the color of the cell.

DHD binding cooperativity

The activation of each node involves previously characterized cooperative binding among DHDs (*32*). Briefly, the system is tuned such that the free energy required to uncage the intramolecular interaction between genetically fused DHD domains (e.g., DHD constructs fused to N-nodes) exceeds the free energy gained by forming one pair of intermolecular heterodimers. In this regime, individual monomers cannot bind to fused DHD domains. The free energy required to uncage the fused DHDs also needs to be smaller than the sum of the free energy gained by forming two matched pairs of heterodimers so the full trimeric complex can form. Previously measured experimental data (*32*) indicate that the cooperative binding can be treated as a two step process. In the first step, one heterodimer binds to the caged DHD, forming a transient dimer with a large off rate. This dimer can be stabilized upon binding to the additional DHD, forming a more stable trimer complex.

Deterministic simulation of the winner-take-all neural network

We performed numerical simulations in Python to gain insights for the behavior of the circuit. Four types of interactions were modeled: protein synthesis, protein binding, protease cleavage, and first-order protein degradation. Here we present the list of possible chemical reactions in the order in which they occur (Fig. 2A).

- Protein synthesis: X_i, N_{ij}^D, C_k denote the DHD inputs, N-half proteases, and C-half proteases, respectively. In the 2-input, 2-output system, they comprise 8 species. The superscript D denotes an attached degron (from DHFR). The subscripts i and j denote the identity of the DHD and protease halves, respectively.

$$\varnothing \rightarrow X_i$$
$$\varnothing \rightarrow N_{ij}^D$$
$$\varnothing \rightarrow C_k$$

- Protein binding (i): Here the DHD, N-half, and C-half protease bind cooperatively to reconstitute the protease complex $C_kX_iN_{ij}^D$ (Fig. 2B). These complexes are active only when j = k, otherwise they represent inactive hybrids, containing mismatched halves of different proteases.

$$X_i + N_{ij}^D \rightarrow X_iN_{ij}^D$$
$$C_k + X_iN_{ij}^D \rightarrow C_kX_iN_{ij}^D$$

- Protease cleavage (i), self-activation: active proteases remove the DHFR tags off proteases of the same kind. $C_kX_iN_{ij}$ is a reconstituted protease without the DHFR degradation domain. It is an active protease when j = k. Otherwise, it represents an inactive hybrid containing protease halves from different proteases. N_{ij} is the N-half protease without the DHFR degradation domain.

Proteases cleave trimers:

$$C_kX_iN_{ij}^D + C_nX_lN_{lm}^D \rightarrow C_kX_iN_{ij}^D + C_nX_lN_{lm} \ (j = k = m)$$
$$C_kX_iN_{ij} + C_nX_lN_{lm}^D \rightarrow C_kX_iN_{ij} + C_nX_lN_{lm} \ (j = k = m)$$

Proteases cleave dimers:

$$C_kX_iN_{ij}^D + X_lN_{lm}^D \rightarrow C_kX_iN_{ij}^D + X_lN_{lm} \ (j = k = m)$$
$$C_kX_iN_{ij} + X_lN_{lm}^D \rightarrow C_kX_iN_{ij} + X_lN_{lm} \ (j = k = m)$$

Proteases cleave monomers:

$$C_kX_iN_{ij}^D + N_{lm}^D \rightarrow C_kX_iN_{ij}^D + N_{lm} \ (j = k = m)$$
$$C_kX_iN_{ij} + N_{lm}^D \rightarrow C_kX_iN_{ij} + N_{lm} \ (j = k = m)$$

- Protease cleavage (ii), mutual inhibition: active proteases cleave the C-half protease of different kinds off their DHD domains. Here C_n^E is a DHD domain without an attached C-half protease (E for empty). $C_n^E X_l N_{lm}^D$ is a protease complex with the DHFR tag and without the C-half protease. $C_n^E X_l N_{lm}$ is a protease complex without the DHFR tag and without the C-half protease.

Proteases cleave monomers:

$$C_k X_i N_{ij}^D + C_n \rightarrow C_k X_i N_{ij}^D + C_n^E \ (j = k \neq n)$$
$$C_k X_i N_{ij} + C_n \rightarrow C_k X_i N_{ij}^D + C_n^E \ (j = k \neq n)$$

Proteases cleave trimers:

$$C_k X_i N_{ij}^D + C_n X_l N_{lm}^D \rightarrow C_k X_i N_{ij}^D + C_n^E X_l N_{lm}^D \ (j = k \neq n)$$
$$C_k X_i N_{ij} + C_n X_l N_{lm}^D \rightarrow C_k X_i N_{ij} + C_n^E X_l N_{lm}^D \ (j = k \neq n)$$

Proteases cleave trimers:

$$C_k X_i N_{ij}^D + C_n X_l N_{lm} \rightarrow C_k X_i N_{ij}^D + C_n^E X_l N_{lm} \ (j = k \neq n)$$
$$C_k X_i N_{ij} + C_n X_l N_{lm} \rightarrow C_k X_i N_{ij} + C_n^E X_l N_{lm} \ (j = k \neq n)$$

- Protein binding (ii): newly formed proteins such as N_{ij} and C_k^E can now participate in binding. $C_k^E X_i N_{ij}^D$ is a protease complex with the DHFR tag and without the C-half protease. $C_k^E X_i N_{ij}$ is a protease complex without the DHFR tag and without the C-half protease. Both complexes are inactive due to the missing C-half protease. In contrast, $C_k X_i N_{ij}$ is an active protease when j = k.

$$X_i + N_{ij} \rightarrow X_i N_{ij}$$
$$X_i N_{ij} + C_k \rightarrow C_k X_i N_{ij}$$
$$X_i N_{ij} + C_k^E \rightarrow C_k^E X_i N_{ij}$$
$$X_i N_{ij}^D + C_k^E \rightarrow C_k^E X_i N_{ij}^D$$

- Protease cleavage (iii): $C_n^E X_l N_{lm}^D$ is a new substrate for protease cleavage.

$$C_k X_i N_{ij}^D + C_n^E X_l N_{lm}^D \rightarrow C_k X_i N_{ij}^D + C_n^E X_l N_{lm} \ (j = k = m)$$
$$C_k X_i N_{ij} + C_n^E X_l N_{lm}^D \rightarrow C_k X_i N_{ij} + C_n^E X_l N_{lm} \ (j = k = m)$$

- Protein degradation: every species with a superscript D, indicating it contains a degron, is assumed to undergo faster degradation than the corresponding species without the superscript D (lacking the degron).

Given the large number of chemical reactions in the system, we wrote a Python script that automatically and programmably generates these reactions and their corresponding ordinary differential equations (ODEs), using reaction rates provided in Table S1. This system of ODEs was then solved using the odeint solver in Python.

Sensitivity analysis

To determine how the classification ability of the winner-take-all neural network depends on various parameters, without loss of generality we fixed the synthesis rates of protein components, while individually varying the catalytic activities of two proteases, the regular degradation rate, and the accelerated degradation rate. The system ordinary differential equations were then solved in Python to obtain final protein species concentrations.

Stochastic simulation of the winner-take-all neural network

To perform stochastic simulations using the Gillespie algorithm (*47*), we first converted mass action rate constants (k) to stochastic rate constants (c) using the following formulae:

0th order reactions: $c = N_A*V*k$
1st order reactions: $c = k$
2nd order reactions: $c = k/(N_A*V)$

where N_A is the Avogadro constant and V is the cell volume. The resulting list of chemical reactions were simulated in Julia (*48*).

Supplementary Text

Sensitivity analysis. At lower values of the protease cleavage catalytic rate, k_{cat}, self-activation and cross-inhibition were hindered, decreasing classification performance. By contrast, when k_{cat} values for both proteases were above 0.04 sec^{-1}, the circuit operated correctly, identifying the larger of the two inputs (Fig. S2C). Higher protein degradation rates, corresponding to stronger degrons in the experimental circuit, suppressed background protease activity, amplifying the effects of self-activation, and generating more pronounced winner-take-all behaviors (Fig. S2D, x-axis).

A list of chemical reaction networks. Here we list all 310 chemical reactions that fully describe a 2-input comparator. “-” indicates an enzyme-substrate complex.

$$X_1 + {N_{11}}^D \underset{koff_1}{\overset{kon_1}{\rightleftharpoons}} X_1{N_{11}}^D \tag{1}$$

$$X_1 + {N_{12}}^D \underset{koff_1}{\overset{kon_1}{\rightleftharpoons}} X_1{N_{12}}^D \tag{2}$$

$$X_2 + {N_{21}}^D \underset{koff_1}{\overset{kon_1}{\rightleftharpoons}} X_2{N_{21}}^D \tag{3}$$

$$X_2 + {N_{22}}^D \underset{koff_1}{\overset{kon_1}{\rightleftharpoons}} X_2{N_{22}}^D \tag{4}$$

$$C_1 + X_1{N_{11}}^D \underset{koff_2}{\overset{kon_2}{\rightleftharpoons}} C_1X_1{N_{11}}^D \tag{5}$$

$$C_1 + X_1{N_{12}}^D \underset{koff_2}{\overset{kon_2}{\rightleftharpoons}} C_1X_1{N_{12}}^D \tag{6}$$

$$C_1 + X_2{N_{21}}^D \underset{koff_2}{\overset{kon_2}{\rightleftharpoons}} C_1X_2{N_{21}}^D \tag{7}$$

$$C_1 + X_2{N_{22}}^D \underset{koff_2}{\overset{kon_2}{\rightleftharpoons}} C_1X_2{N_{22}}^D \tag{8}$$

$$C_2 + X_1{N_{11}}^D \underset{koff_2}{\overset{kon_2}{\rightleftharpoons}} C_2X_1{N_{11}}^D \tag{9}$$

$$C_2 + X_1{N_{12}}^D \underset{koff_2}{\overset{kon_2}{\rightleftharpoons}} C_2X_1{N_{12}}^D \tag{10}$$

$$C_2 + X_2{N_{21}}^D \underset{koff_2}{\overset{kon_2}{\rightleftharpoons}} C_2X_2{N_{21}}^D \tag{11}$$

$$C_2 + X_2{N_{22}}^D \underset{koff_2}{\overset{kon_2}{\rightleftharpoons}} C_2X_2{N_{22}}^D \tag{12}$$

$$C_1X_1{N_{11}}^D + {N_{11}}^D \underset{koff_{p1}}{\overset{kon_{p1}}{\rightleftharpoons}} C_1X_1{N_{11}}^D{-}{N_{11}}^D \tag{13}$$

$$C_1X_1{N_{11}}^D + {N_{21}}^D \underset{koff_{p1}}{\overset{kon_{p1}}{\rightleftharpoons}} C_1X_1{N_{11}}^D{-}{N_{21}}^D \tag{14}$$

$$C_1X_2{N_{21}}^D + {N_{11}}^D \underset{koff_{p1}}{\overset{kon_{p1}}{\rightleftharpoons}} C_1X_2{N_{21}}^D{-}{N_{11}}^D \tag{15}$$

$$C_1X_2{N_{21}}^D + {N_{21}}^D \underset{koff_{p1}}{\overset{kon_{p1}}{\rightleftharpoons}} C_1X_2{N_{21}}^D{-}{N_{21}}^D \tag{16}$$

$$C_2X_1{N_{12}}^D + {N_{12}}^D \underset{koff_{p2}}{\overset{kon_{p2}}{\rightleftharpoons}} C_2X_1{N_{12}}^D{-}{N_{12}}^D \tag{17}$$

$$C_2X_1{N_{12}}^D + {N_{22}}^D \underset{koff_{p2}}{\overset{kon_{p2}}{\rightleftharpoons}} C_2X_1{N_{12}}^D{-}{N_{22}}^D \tag{18}$$

$$C_2X_2{N_{22}}^D + {N_{12}}^D \underset{koff_{p2}}{\overset{kon_{p2}}{\rightleftharpoons}} C_2X_2{N_{22}}^D{-}{N_{12}}^D \tag{19}$$

$$C_2X_2{N_{22}}^D + {N_{22}}^D \underset{koff_{p2}}{\overset{kon_{p2}}{\rightleftharpoons}} C_2X_2{N_{22}}^D{-}{N_{22}}^D \tag{20}$$

$$C_1X_1{N_{11}}^D{-}{N_{11}}^D \xrightarrow{kcat_1} C_1X_1{N_{11}}^D + N_{11} \tag{21}$$

$$C_1X_1{N_{11}}^D{-}{N_{21}}^D \xrightarrow{kcat_1} C_1X_1{N_{11}}^D + N_{21} \tag{22}$$

$$C_1X_2{N_{21}}^D{-}{N_{11}}^D \xrightarrow{kcat_1} C_1X_2{N_{21}}^D + N_{11} \tag{23}$$

$$C_1X_2{N_{21}}^D{-}{N_{21}}^D \xrightarrow{kcat_1} C_1X_2{N_{21}}^D + N_{21} \tag{24}$$

$$C_2X_1{N_{12}}^D{-}{N_{12}}^D \xrightarrow{kcat_2} C_2X_1{N_{12}}^D + N_{12} \tag{25}$$

$$C_2X_1N_{12}{}^{D}{-}N_{22}{}^{D} \xrightarrow{kcat_2} C_2X_1N_{12}{}^{D} + N_{22} \tag{26}$$

$$C_2X_2N_{22}{}^{D}{-}N_{12}{}^{D} \xrightarrow{kcat_2} C_2X_2N_{22}{}^{D} + N_{12} \tag{27}$$

$$C_2X_2N_{22}{}^{D}{-}N_{22}{}^{D} \xrightarrow{kcat_2} C_2X_2N_{22}{}^{D} + N_{22} \tag{28}$$

$$C_1X_1N_{11}{}^{D} + X_1N_{11}{}^{D} \underset{koff_{p1}}{\overset{kon_{p1}}{\rightleftharpoons}} C_1X_1N_{11}{}^{D}{-}X_1N_{11}{}^{D} \tag{29}$$

$$C_1X_1N_{11}{}^{D} + X_2N_{21}{}^{D} \underset{koff_{p1}}{\overset{kon_{p1}}{\rightleftharpoons}} C_1X_1N_{11}{}^{D}{-}X_2N_{21}{}^{D} \tag{30}$$

$$C_1X_2N_{21}{}^{D} + X_1N_{11}{}^{D} \underset{koff_{p1}}{\overset{kon_{p1}}{\rightleftharpoons}} C_1X_2N_{21}{}^{D}{-}X_1N_{11}{}^{D} \tag{31}$$

$$C_1X_2N_{21}{}^{D} + X_2N_{21}{}^{D} \underset{koff_{p1}}{\overset{kon_{p1}}{\rightleftharpoons}} C_1X_2N_{21}{}^{D}{-}X_2N_{21}{}^{D} \tag{32}$$

$$C_2X_1N_{12}{}^{D} + X_1N_{12}{}^{D} \underset{koff_{p2}}{\overset{kon_{p2}}{\rightleftharpoons}} C_2X_1N_{12}{}^{D}{-}X_1N_{12}{}^{D} \tag{33}$$

$$C_2X_1N_{12}{}^{D} + X_2N_{22}{}^{D} \underset{koff_{p2}}{\overset{kon_{p2}}{\rightleftharpoons}} C_2X_1N_{12}{}^{D}{-}X_2N_{22}{}^{D} \tag{34}$$

$$C_2X_2N_{22}{}^{D} + X_1N_{12}{}^{D} \underset{koff_{p2}}{\overset{kon_{p2}}{\rightleftharpoons}} C_2X_2N_{22}{}^{D}{-}X_1N_{12}{}^{D} \tag{35}$$

$$C_2X_2N_{22}{}^{D} + X_2N_{22}{}^{D} \underset{koff_{p2}}{\overset{kon_{p2}}{\rightleftharpoons}} C_2X_2N_{22}{}^{D}{-}X_2N_{22}{}^{D} \tag{36}$$

$$C_1X_1N_{11}{}^{D}{-}X_1N_{11}{}^{D} \xrightarrow{kcat_1} C_1X_1N_{11}{}^{D} + X_1N_{11} \tag{37}$$

$$C_1X_1N_{11}{}^{D}{-}X_2N_{21}{}^{D} \xrightarrow{kcat_1} C_1X_1N_{11}{}^{D} + X_2N_{21} \tag{38}$$

$$C_1X_2N_{21}{}^{D}{-}X_1N_{11}{}^{D} \xrightarrow{kcat_1} C_1X_2N_{21}{}^{D} + X_1N_{11} \tag{39}$$

$$C_1X_2N_{21}{}^{D}{-}X_2N_{21}{}^{D} \xrightarrow{kcat_1} C_1X_2N_{21}{}^{D} + X_2N_{21} \tag{40}$$

$$C_2X_1N_{12}{}^{D}{-}X_1N_{12}{}^{D} \xrightarrow{kcat_2} C_2X_1N_{12}{}^{D} + X_1N_{12} \tag{41}$$

$$C_2X_1N_{12}{}^{D}{-}X_2N_{22}{}^{D} \xrightarrow{kcat_2} C_2X_1N_{12}{}^{D} + X_2N_{22} \tag{42}$$

$$C_2X_2N_{22}{}^{D}{-}X_1N_{12}{}^{D} \xrightarrow{kcat_2} C_2X_2N_{22}{}^{D} + X_1N_{12} \tag{43}$$

$$C_2X_2N_{22}{}^{D}{-}X_2N_{22}{}^{D} \xrightarrow{kcat_2} C_2X_2N_{22}{}^{D} + X_2N_{22} \tag{44}$$

$$2\,C_1X_1N_{11}{}^{D} \underset{koff_{p1}}{\overset{kon_{p1}}{\rightleftharpoons}} C_1X_1N_{11}{}^{D}{-}C_1X_1N_{11}{}^{D} \tag{45}$$

$$C_1X_1N_{11}{}^{D} + C_1X_2N_{21}{}^{D} \underset{koff_{p1}}{\overset{kon_{p1}}{\rightleftharpoons}} C_1X_1N_{11}{}^{D}{-}C_1X_2N_{21}{}^{D} \tag{46}$$

$$C_1X_1N_{11}{}^{D} + C_2X_1N_{11}{}^{D} \underset{koff_{p1}}{\overset{kon_{p1}}{\rightleftharpoons}} C_1X_1N_{11}{}^{D}{-}C_2X_1N_{11}{}^{D} \tag{47}$$

$$C_1X_1N_{11}{}^{D} + C_2X_2N_{21}{}^{D} \underset{koff_{p1}}{\overset{kon_{p1}}{\rightleftharpoons}} C_1X_1N_{11}{}^{D}{-}C_2X_2N_{21}{}^{D} \tag{48}$$

$$C_1X_2N_{21}{}^{D} + C_1X_1N_{11}{}^{D} \underset{koff_{p1}}{\overset{kon_{p1}}{\rightleftharpoons}} C_1X_2N_{21}{}^{D}{-}C_1X_1N_{11}{}^{D} \tag{49}$$

$$2\,C_1X_2N_{21}{}^{D} \underset{koff_{p1}}{\overset{kon_{p1}}{\rightleftharpoons}} C_1X_2N_{21}{}^{D}{-}C_1X_2N_{21}{}^{D} \tag{50}$$

$$C_1X_2N_{21}{}^{D} + C_2X_1N_{11}{}^{D} \underset{koff_{p1}}{\overset{kon_{p1}}{\rightleftharpoons}} C_1X_2N_{21}{}^{D}{-}C_2X_1N_{11}{}^{D} \tag{51}$$

$$C_1X_2N_{21}{}^{D} + C_2X_2N_{21}{}^{D} \underset{koff_{p1}}{\overset{kon_{p1}}{\rightleftharpoons}} C_1X_2N_{21}{}^{D}{-}C_2X_2N_{21}{}^{D} \tag{52}$$

$$C_2X_1N_{12}{}^{D} + C_1X_1N_{12}{}^{D} \underset{koff_{p2}}{\overset{kon_{p2}}{\rightleftharpoons}} C_2X_1N_{12}{}^{D}{-}C_1X_1N_{12}{}^{D} \tag{53}$$

$$C_2X_1N_{12}{}^{D} + C_1X_2N_{22}{}^{D} \underset{koff_{p2}}{\overset{kon_{p2}}{\rightleftharpoons}} C_2X_1N_{12}{}^{D}{-}C_1X_2N_{22}{}^{D} \tag{54}$$

$$2\,C_2X_1N_{12}{}^{D} \underset{koff_{p2}}{\overset{kon_{p2}}{\rightleftharpoons}} C_2X_1N_{12}{}^{D}{-}C_2X_1N_{12}{}^{D} \tag{55}$$

$$C_2X_1N_{12}{}^{D} + C_2X_2N_{22}{}^{D} \underset{koff_{p2}}{\overset{kon_{p2}}{\rightleftharpoons}} C_2X_1N_{12}{}^{D}{-}C_2X_2N_{22}{}^{D} \tag{56}$$

$$C_2X_2N_{22}{}^{D} + C_1X_1N_{12}{}^{D} \underset{koff_{p2}}{\overset{kon_{p2}}{\rightleftharpoons}} C_2X_2N_{22}{}^{D}{-}C_1X_1N_{12}{}^{D} \tag{57}$$

$$C_2X_2N_{22}{}^{D} + C_1X_2N_{22}{}^{D} \underset{koff_{p2}}{\overset{kon_{p2}}{\rightleftharpoons}} C_2X_2N_{22}{}^{D}{-}C_1X_2N_{22}{}^{D} \tag{58}$$

$$C_2X_2N_{22}{}^{D} + C_2X_1N_{12}{}^{D} \underset{koff_{p2}}{\overset{kon_{p2}}{\rightleftharpoons}} C_2X_2N_{22}{}^{D}{-}C_2X_1N_{12}{}^{D} \tag{59}$$

$$2\,C_2X_2N_{22}{}^{D} \underset{koff_{p2}}{\overset{kon_{p2}}{\rightleftharpoons}} C_2X_2N_{22}{}^{D}{-}C_2X_2N_{22}{}^{D} \tag{60}$$

$$C_1X_1N_{11}{}^{D}{-}C_1X_1N_{11}{}^{D} \xrightarrow{kcat_1} C_1X_1N_{11}{}^{D} + C_1X_1N_{11} \tag{61}$$

$$C_1X_1N_{11}{}^{D}{-}C_1X_2N_{21}{}^{D} \xrightarrow{kcat_1} C_1X_1N_{11}{}^{D} + C_1X_2N_{21} \tag{62}$$

$$C_1X_1N_{11}{}^{D}{-}C_2X_1N_{11}{}^{D} \xrightarrow{kcat_1} C_1X_1N_{11}{}^{D} + C_2X_1N_{11} \tag{63}$$

$$C_1X_1N_{11}{}^{D}{-}C_2X_2N_{21}{}^{D} \xrightarrow{kcat_1} C_1X_1N_{11}{}^{D} + C_2X_2N_{21} \tag{64}$$

$$C_1X_2N_{21}{}^{D}{-}C_1X_1N_{11}{}^{D} \xrightarrow{kcat_1} C_1X_2N_{21}{}^{D} + C_1X_1N_{11} \tag{65}$$

$$C_1X_2N_{21}{}^{D}{-}C_1X_2N_{21}{}^{D} \xrightarrow{kcat_1} C_1X_2N_{21}{}^{D} + C_1X_2N_{21} \tag{66}$$

$$C_1X_2N_{21}{}^{D}{-}C_2X_1N_{11}{}^{D} \xrightarrow{kcat_1} C_1X_2N_{21}{}^{D} + C_2X_1N_{11} \tag{67}$$

$$C_1X_2N_{21}{}^{D}{-}C_2X_2N_{21}{}^{D} \xrightarrow{kcat_1} C_1X_2N_{21}{}^{D} + C_2X_2N_{21} \tag{68}$$

$$C_2X_1N_{12}{}^{D}{-}C_1X_1N_{12}{}^{D} \xrightarrow{kcat_2} C_2X_1N_{12}{}^{D} + C_1X_1N_{12} \tag{69}$$

$$C_2X_1N_{12}{}^{D}{-}C_1X_2N_{22}{}^{D} \xrightarrow{kcat_2} C_2X_1N_{12}{}^{D} + C_1X_2N_{22} \tag{70}$$

$$C_2X_1N_{12}{}^{D}{-}C_2X_1N_{12}{}^{D} \xrightarrow{kcat_2} C_2X_1N_{12}{}^{D} + C_2X_1N_{12} \tag{71}$$

$$C_2X_1N_{12}{}^{D}{-}C_2X_2N_{22}{}^{D} \xrightarrow{kcat_2} C_2X_1N_{12}{}^{D} + C_2X_2N_{22} \tag{72}$$

$$C_2X_2N_{22}{}^{D}{-}C_1X_1N_{12}{}^{D} \xrightarrow{kcat_2} C_2X_2N_{22}{}^{D} + C_1X_1N_{12} \tag{73}$$

$$C_2X_2N_{22}{}^{D}{-}C_1X_2N_{22}{}^{D} \xrightarrow{kcat_2} C_2X_2N_{22}{}^{D} + C_1X_2N_{22} \tag{74}$$

$$C_2X_2N_{22}{}^{D}{-}C_2X_1N_{12}{}^{D} \xrightarrow{kcat_2} C_2X_2N_{22}{}^{D} + C_2X_1N_{12} \tag{75}$$

$$C_2X_2N_{22}{}^{D}{-}C_2X_2N_{22}{}^{D} \xrightarrow{kcat_2} C_2X_2N_{22}{}^{D} + C_2X_2N_{22} \tag{76}$$

$$C_1X_1N_{11}{}^{D} + C_2 \underset{koff_{p1}}{\overset{kon_{p1}}{\rightleftharpoons}} C_1X_1N_{11}{}^{D}{-}C_2 \tag{77}$$

$$C_1X_2N_{21}{}^{D} + C_2 \underset{koff_{p1}}{\overset{kon_{p1}}{\rightleftharpoons}} C_1X_2N_{21}{}^{D}{-}C_2 \tag{78}$$

$$C_2X_1N_{12}{}^{D} + C_1 \underset{koff_{p2}}{\overset{kon_{p2}}{\rightleftharpoons}} C_2X_1N_{12}{}^{D}{-}C_1 \tag{79}$$

$$C_2X_2N_{22}{}^{D} + C_1 \underset{koff_{p2}}{\overset{kon_{p2}}{\rightleftharpoons}} C_2X_2N_{22}{}^{D}{-}C_1 \tag{80}$$

$$C_1X_1N_{11}{}^{D}{-}C_2 \xrightarrow{kcat_1} C_1X_1N_{11}{}^{D} + C_2{}^{E} \tag{81}$$

$$C_1X_2N_{21}{}^{D}{-}C_2 \xrightarrow{kcat_1} C_1X_2N_{21}{}^{D} + C_2{}^{E} \tag{82}$$

$$C_2X_1N_{12}{}^{D}{-}C_1 \xrightarrow{kcat_2} C_2X_1N_{12}{}^{D} + C_1{}^{E} \tag{83}$$

$$C_2X_2N_{22}{}^{D}{-}C_1 \xrightarrow{kcat_2} C_2X_2N_{22}{}^{D} + C_1{}^{E} \tag{84}$$

$$X_1 + N_{11} \underset{koff_1}{\overset{kon_1}{\rightleftharpoons}} X_1N_{11} \tag{85}$$

$$X_1 + N_{12} \underset{koff_1}{\overset{kon_1}{\rightleftharpoons}} X_1N_{12} \tag{86}$$

$$X_2 + N_{21} \underset{koff_1}{\overset{kon_1}{\rightleftharpoons}} X_2N_{21} \tag{87}$$

$$X_2 + N_{22} \underset{koff_1}{\overset{kon_1}{\rightleftharpoons}} X_2N_{22} \tag{88}$$

$$C_1 + X_1N_{11} \underset{koff_2}{\overset{kon_2}{\rightleftharpoons}} C_1X_1N_{11} \tag{89}$$

$$C_1 + X_1N_{12} \underset{koff_2}{\overset{kon_2}{\rightleftharpoons}} C_1X_1N_{12} \tag{90}$$

$$C_1 + X_2N_{21} \underset{koff_2}{\overset{kon_2}{\rightleftharpoons}} C_1X_2N_{21} \tag{91}$$

$$C_1 + X_2N_{22} \underset{koff_2}{\overset{kon_2}{\rightleftharpoons}} C_1X_2N_{22} \tag{92}$$

$$C_2 + X_1N_{11} \underset{koff_2}{\overset{kon_2}{\rightleftharpoons}} C_2X_1N_{11} \tag{93}$$

$$C_2 + X_1N_{12} \underset{koff_2}{\overset{kon_2}{\rightleftharpoons}} C_2X_1N_{12} \tag{94}$$

$$C_2 + X_2N_{21} \underset{koff_2}{\overset{kon_2}{\rightleftharpoons}} C_2X_2N_{21} \tag{95}$$

$$C_2 + X_2N_{22} \underset{koff_2}{\overset{kon_2}{\rightleftharpoons}} C_2X_2N_{22} \tag{96}$$

$$C_1X_1N_{11} + N_{11}{}^{D} \underset{koff_{p1}}{\overset{kon_{p1}}{\rightleftharpoons}} C_1X_1N_{11}{-}N_{11}{}^{D} \tag{97}$$

$$C_1X_1N_{11} + N_{21}{}^{D} \underset{koff_{p1}}{\overset{kon_{p1}}{\rightleftharpoons}} C_1X_1N_{11}{-}N_{21}{}^{D} \tag{98}$$

$$C_1X_2N_{21} + N_{11}{}^{D} \underset{koff_{p1}}{\overset{kon_{p1}}{\rightleftharpoons}} C_1X_2N_{21}{-}N_{11}{}^{D} \tag{99}$$

$$C_1X_2N_{21} + N_{21}{}^{D} \underset{koff_{p1}}{\overset{kon_{p1}}{\rightleftharpoons}} C_1X_2N_{21}{-}N_{21}{}^{D} \tag{100}$$

$$C_2X_1N_{12} + N_{12}{}^{D} \underset{koff_{p2}}{\overset{kon_{p2}}{\rightleftharpoons}} C_2X_1N_{12}{-}N_{12}{}^{D} \tag{101}$$

$$C_2X_1N_{12} + N_{22}{}^{D} \underset{koff_{p2}}{\overset{kon_{p2}}{\rightleftharpoons}} C_2X_1N_{12}{-}N_{22}{}^{D} \tag{102}$$

$$C_2X_2N_{22} + N_{12}{}^{D} \underset{koff_{p2}}{\overset{kon_{p2}}{\rightleftharpoons}} C_2X_2N_{22}{-}N_{12}{}^{D} \tag{103}$$

$$C_2X_2N_{22} + N_{22}{}^{D} \underset{koff_{p2}}{\overset{kon_{p2}}{\rightleftharpoons}} C_2X_2N_{22}{-}N_{22}{}^{D} \tag{104}$$

$$C_1X_1N_{11}{-}N_{11}{}^{D} \xrightarrow{kcat_1} C_1X_1N_{11} + N_{11} \tag{105}$$

$$C_1X_1N_{11}{-}N_{21}{}^{D} \xrightarrow{kcat_1} C_1X_1N_{11} + N_{21} \tag{106}$$

$$C_1X_2N_{21}{-}N_{11}{}^{D} \xrightarrow{kcat_1} C_1X_2N_{21} + N_{11} \tag{107}$$

$$C_1X_2N_{21}{-}N_{21}{}^{D} \xrightarrow{kcat_1} C_1X_2N_{21} + N_{21} \tag{108}$$

$$C_2X_1N_{12}{-}N_{12}{}^{D} \xrightarrow{kcat_2} C_2X_1N_{12} + N_{12} \tag{109}$$

$$C_2X_1N_{12}{-}N_{22}{}^{D} \xrightarrow{kcat_2} C_2X_1N_{12} + N_{22} \tag{110}$$

$$C_2X_2N_{22}{-}N_{12}{}^{D} \xrightarrow{kcat_2} C_2X_2N_{22} + N_{12} \tag{111}$$

$$C_2X_2N_{22}{-}N_{22}{}^{D} \xrightarrow{kcat_2} C_2X_2N_{22} + N_{22} \tag{112}$$

$$C_1X_1N_{11} + X_1N_{11}{}^{D} \underset{koff_{p1}}{\overset{kon_{p1}}{\rightleftharpoons}} C_1X_1N_{11}{-}X_1N_{11}{}^{D} \tag{113}$$

$$C_1X_1N_{11} + X_2N_{21}{}^{D} \underset{koff_{p1}}{\overset{kon_{p1}}{\rightleftharpoons}} C_1X_1N_{11}{-}X_2N_{21}{}^{D} \tag{114}$$

$$C_1X_2N_{21} + X_1N_{11}{}^{D} \underset{koff_{p1}}{\overset{kon_{p1}}{\rightleftharpoons}} C_1X_2N_{21}{-}X_1N_{11}{}^{D} \tag{115}$$

$$C_1X_2N_{21} + X_2N_{21}{}^{D} \underset{koff_{p1}}{\overset{kon_{p1}}{\rightleftharpoons}} C_1X_2N_{21}{-}X_2N_{21}{}^{D} \tag{116}$$

$$C_2X_1N_{12} + X_1N_{12}{}^{D} \underset{koff_{p2}}{\overset{kon_{p2}}{\rightleftharpoons}} C_2X_1N_{12}{-}X_1N_{12}{}^{D} \tag{117}$$

$$C_2X_1N_{12} + X_2N_{22}{}^{D} \underset{koff_{p2}}{\overset{kon_{p2}}{\rightleftharpoons}} C_2X_1N_{12}{-}X_2N_{22}{}^{D} \tag{118}$$

$$C_2X_2N_{22} + X_1N_{12}{}^{D} \underset{koff_{p2}}{\overset{kon_{p2}}{\rightleftharpoons}} C_2X_2N_{22}{-}X_1N_{12}{}^{D} \tag{119}$$

$$C_2X_2N_{22} + X_2N_{22}{}^{D} \underset{koff_{p2}}{\overset{kon_{p2}}{\rightleftharpoons}} C_2X_2N_{22}{-}X_2N_{22}{}^{D} \tag{120}$$

$$C_1X_1N_{11}{-}X_1N_{11}{}^{D} \xrightarrow{kcat_1} C_1X_1N_{11} + X_1N_{11} \tag{121}$$

$$C_1X_1N_{11}{-}X_2N_{21}{}^{D} \xrightarrow{kcat_1} C_1X_1N_{11} + X_2N_{21} \tag{122}$$

$$C_1X_2N_{21}{-}X_1N_{11}{}^{D} \xrightarrow{kcat_1} C_1X_2N_{21} + X_1N_{11} \tag{123}$$

$$C_1X_2N_{21}{-}X_2N_{21}{}^{D} \xrightarrow{kcat_1} C_1X_2N_{21} + X_2N_{21} \tag{124}$$

$$C_2X_1N_{12}{-}X_1N_{12}{}^{D} \xrightarrow{kcat_2} C_2X_1N_{12} + X_1N_{12} \tag{125}$$

$$C_2X_1N_{12}{-}X_2N_{22}{}^D \xrightarrow{kcat_2} C_2X_1N_{12} + X_2N_{22} \tag{126}$$

$$C_2X_2N_{22}{-}X_1N_{12}{}^D \xrightarrow{kcat_2} C_2X_2N_{22} + X_1N_{12} \tag{127}$$

$$C_2X_2N_{22}{-}X_2N_{22}{}^D \xrightarrow{kcat_2} C_2X_2N_{22} + X_2N_{22} \tag{128}$$

$$C_1X_1N_{11} + C_1X_1N_{11}{}^D \underset{koff_{p1}}{\overset{kon_{p1}}{\rightleftharpoons}} C_1X_1N_{11}{-}C_1X_1N_{11}{}^D \tag{129}$$

$$C_1X_1N_{11} + C_1X_2N_{21}{}^D \underset{koff_{p1}}{\overset{kon_{p1}}{\rightleftharpoons}} C_1X_1N_{11}{-}C_1X_2N_{21}{}^D \tag{130}$$

$$C_1X_1N_{11} + C_2X_1N_{11}{}^D \underset{koff_{p1}}{\overset{kon_{p1}}{\rightleftharpoons}} C_1X_1N_{11}{-}C_2X_1N_{11}{}^D \tag{131}$$

$$C_1X_1N_{11} + C_2X_2N_{21}{}^D \underset{koff_{p1}}{\overset{kon_{p1}}{\rightleftharpoons}} C_1X_1N_{11}{-}C_2X_2N_{21}{}^D \tag{132}$$

$$C_1X_2N_{21} + C_1X_1N_{11}{}^D \underset{koff_{p1}}{\overset{kon_{p1}}{\rightleftharpoons}} C_1X_2N_{21}{-}C_1X_1N_{11}{}^D \tag{133}$$

$$C_1X_2N_{21} + C_1X_2N_{21}{}^D \underset{koff_{p1}}{\overset{kon_{p1}}{\rightleftharpoons}} C_1X_2N_{21}{-}C_1X_2N_{21}{}^D \tag{134}$$

$$C_1X_2N_{21} + C_2X_1N_{11}{}^D \underset{koff_{p1}}{\overset{kon_{p1}}{\rightleftharpoons}} C_1X_2N_{21}{-}C_2X_1N_{11}{}^D \tag{135}$$

$$C_1X_2N_{21} + C_2X_2N_{21}{}^D \underset{koff_{p1}}{\overset{kon_{p1}}{\rightleftharpoons}} C_1X_2N_{21}{-}C_2X_2N_{21}{}^D \tag{136}$$

$$C_2X_1N_{12} + C_1X_1N_{12}{}^D \underset{koff_{p2}}{\overset{kon_{p2}}{\rightleftharpoons}} C_2X_1N_{12}{-}C_1X_1N_{12}{}^D \tag{137}$$

$$C_2X_1N_{12} + C_1X_2N_{22}{}^D \underset{koff_{p2}}{\overset{kon_{p2}}{\rightleftharpoons}} C_2X_1N_{12}{-}C_1X_2N_{22}{}^D \tag{138}$$

$$C_2X_1N_{12} + C_2X_1N_{12}{}^D \underset{koff_{p2}}{\overset{kon_{p2}}{\rightleftharpoons}} C_2X_1N_{12}{-}C_2X_1N_{12}{}^D \tag{139}$$

$$C_2X_1N_{12} + C_2X_2N_{22}{}^D \underset{koff_{p2}}{\overset{kon_{p2}}{\rightleftharpoons}} C_2X_1N_{12}{-}C_2X_2N_{22}{}^D \tag{140}$$

$$C_2X_2N_{22} + C_1X_1N_{12}{}^D \underset{koff_{p2}}{\overset{kon_{p2}}{\rightleftharpoons}} C_2X_2N_{22}{-}C_1X_1N_{12}{}^D \tag{141}$$

$$C_2X_2N_{22} + C_1X_2N_{22}{}^D \underset{koff_{p2}}{\overset{kon_{p2}}{\rightleftharpoons}} C_2X_2N_{22}{-}C_1X_2N_{22}{}^D \tag{142}$$

$$C_2X_2N_{22} + C_2X_1N_{12}{}^D \underset{koff_{p2}}{\overset{kon_{p2}}{\rightleftharpoons}} C_2X_2N_{22}{-}C_2X_1N_{12}{}^D \tag{143}$$

$$C_2X_2N_{22} + C_2X_2N_{22}{}^D \underset{koff_{p2}}{\overset{kon_{p2}}{\rightleftharpoons}} C_2X_2N_{22}{-}C_2X_2N_{22}{}^D \tag{144}$$

$$C_1X_1N_{11}{-}C_1X_1N_{11}{}^D \xrightarrow{kcat_1} 2\,C_1X_1N_{11} \tag{145}$$

$$C_1X_1N_{11}{-}C_1X_2N_{21}{}^D \xrightarrow{kcat_1} C_1X_1N_{11} + C_1X_2N_{21} \tag{146}$$

$$C_1X_1N_{11}{-}C_2X_1N_{11}{}^D \xrightarrow{kcat_1} C_1X_1N_{11} + C_2X_1N_{11} \tag{147}$$

$$C_1X_1N_{11}{-}C_2X_2N_{21}{}^D \xrightarrow{kcat_1} C_1X_1N_{11} + C_2X_2N_{21} \tag{148}$$

$$C_1X_2N_{21}{-}C_1X_1N_{11}{}^D \xrightarrow{kcat_1} C_1X_2N_{21} + C_1X_1N_{11} \tag{149}$$

$$C_1X_2N_{21}{-}C_1X_2N_{21}{}^D \xrightarrow{kcat_1} 2\,C_1X_2N_{21} \tag{150}$$

$$C_1X_2N_{21}{-}C_2X_1N_{11}{}^D \xrightarrow{kcat_1} C_1X_2N_{21} + C_2X_1N_{11} \tag{151}$$

$$C_1X_2N_{21}{-}C_2X_2N_{21}{}^D \xrightarrow{kcat_1} C_1X_2N_{21} + C_2X_2N_{21} \tag{152}$$

$$C_2X_1N_{12}{-}C_1X_1N_{12}{}^D \xrightarrow{kcat_2} C_2X_1N_{12} + C_1X_1N_{12} \tag{153}$$

$$C_2X_1N_{12}{-}C_1X_2N_{22}{}^D \xrightarrow{kcat_2} C_2X_1N_{12} + C_1X_2N_{22} \tag{154}$$

$$C_2X_1N_{12}{-}C_2X_1N_{12}{}^D \xrightarrow{kcat_2} 2\,C_2X_1N_{12} \tag{155}$$

$$C_2X_1N_{12}{-}C_2X_2N_{22}{}^D \xrightarrow{kcat_2} C_2X_1N_{12} + C_2X_2N_{22} \tag{156}$$

$$C_2X_2N_{22}{-}C_1X_1N_{12}{}^D \xrightarrow{kcat_2} C_2X_2N_{22} + C_1X_1N_{12} \tag{157}$$

$$C_2X_2N_{22}{-}C_1X_2N_{22}{}^D \xrightarrow{kcat_2} C_2X_2N_{22} + C_1X_2N_{22} \tag{158}$$

$$C_2X_2N_{22}{-}C_2X_1N_{12}{}^D \xrightarrow{kcat_2} C_2X_2N_{22} + C_2X_1N_{12} \tag{159}$$

$$C_2X_2N_{22}{-}C_2X_2N_{22}{}^D \xrightarrow{kcat_2} 2\,C_2X_2N_{22} \tag{160}$$

$$C_1X_1N_{11} + C_2 \underset{koff_{p1}}{\overset{kon_{p1}}{\rightleftharpoons}} C_1X_1N_{11}{-}C_2 \tag{161}$$

$$C_1X_2N_{21} + C_2 \underset{koff_{p1}}{\overset{kon_{p1}}{\rightleftharpoons}} C_1X_2N_{21}{-}C_2 \tag{162}$$

$$C_2X_1N_{12} + C_1 \underset{koff_{p2}}{\overset{kon_{p2}}{\rightleftharpoons}} C_2X_1N_{12}{-}C_1 \tag{163}$$

$$C_2X_2N_{22} + C_1 \underset{koff_{p2}}{\overset{kon_{p2}}{\rightleftharpoons}} C_2X_2N_{22}{-}C_1 \tag{164}$$

$$C_1X_1N_{11}{-}C_2 \xrightarrow{kcat_1} C_1X_1N_{11} + C_2{}^E \tag{165}$$

$$C_1X_2N_{21}{-}C_2 \xrightarrow{kcat_1} C_1X_2N_{21} + C_2{}^E \tag{166}$$

$$C_2X_1N_{12}{-}C_1 \xrightarrow{kcat_2} C_2X_1N_{12} + C_1{}^E \tag{167}$$

$$C_2X_2N_{22}{-}C_1 \xrightarrow{kcat_2} C_2X_2N_{22} + C_1{}^E \tag{168}$$

$$C_2{}^E + X_1N_{11}{}^D \underset{koff_2}{\overset{kon_2}{\rightleftharpoons}} C_2{}^EX_1N_{11}{}^D \tag{169}$$

$$C_2{}^E + X_1N_{12}{}^D \underset{koff_2}{\overset{kon_2}{\rightleftharpoons}} C_2{}^EX_1N_{12}{}^D \tag{170}$$

$$C_2{}^E + X_2N_{21}{}^D \underset{koff_2}{\overset{kon_2}{\rightleftharpoons}} C_2{}^EX_2N_{21}{}^D \tag{171}$$

$$C_2{}^E + X_2N_{22}{}^D \underset{koff_2}{\overset{kon_2}{\rightleftharpoons}} C_2{}^EX_2N_{22}{}^D \tag{172}$$

$$C_1{}^E + X_1N_{11}{}^D \underset{koff_2}{\overset{kon_2}{\rightleftharpoons}} C_1{}^EX_1N_{11}{}^D \tag{173}$$

$$C_1{}^E + X_1N_{12}{}^D \underset{koff_2}{\overset{kon_2}{\rightleftharpoons}} C_1{}^EX_1N_{12}{}^D \tag{174}$$

$$C_1{}^E + X_2N_{21}{}^D \underset{koff_2}{\overset{kon_2}{\rightleftharpoons}} C_1{}^EX_2N_{21}{}^D \tag{175}$$

$$C_1{}^E + X_2N_{22}{}^D \underset{\mathrm{koff}_2}{\overset{\mathrm{kon}_2}{\rightleftharpoons}} C_1{}^EX_2N_{22}{}^D \tag{176}$$

$$C_2{}^E + X_1N_{11} \underset{\mathrm{koff}_2}{\overset{\mathrm{kon}_2}{\rightleftharpoons}} C_2{}^EX_1N_{11} \tag{177}$$

$$C_2{}^E + X_2N_{21} \underset{\mathrm{koff}_2}{\overset{\mathrm{kon}_2}{\rightleftharpoons}} C_2{}^EX_2N_{21} \tag{178}$$

$$C_2{}^E + X_1N_{12} \underset{\mathrm{koff}_2}{\overset{\mathrm{kon}_2}{\rightleftharpoons}} C_2{}^EX_1N_{12} \tag{179}$$

$$C_2{}^E + X_2N_{22} \underset{\mathrm{koff}_2}{\overset{\mathrm{kon}_2}{\rightleftharpoons}} C_2{}^EX_2N_{22} \tag{180}$$

$$C_1{}^E + X_1N_{11} \underset{\mathrm{koff}_2}{\overset{\mathrm{kon}_2}{\rightleftharpoons}} C_1{}^EX_1N_{11} \tag{181}$$

$$C_1{}^E + X_2N_{21} \underset{\mathrm{koff}_2}{\overset{\mathrm{kon}_2}{\rightleftharpoons}} C_1{}^EX_2N_{21} \tag{182}$$

$$C_1{}^E + X_1N_{12} \underset{\mathrm{koff}_2}{\overset{\mathrm{kon}_2}{\rightleftharpoons}} C_1{}^EX_1N_{12} \tag{183}$$

$$C_1{}^E + X_2N_{22} \underset{\mathrm{koff}_2}{\overset{\mathrm{kon}_2}{\rightleftharpoons}} C_1{}^EX_2N_{22} \tag{184}$$

$$C_1X_1N_{11}{}^D + C_2{}^EX_1N_{11}{}^D \underset{\mathrm{koff}_{p1}}{\overset{\mathrm{kon}_{p1}}{\rightleftharpoons}} C_1X_1N_{11}{}^D{-}C_2{}^EX_1N_{11}{}^D \tag{185}$$

$$C_1X_1N_{11}{}^D + C_2{}^EX_2N_{21}{}^D \underset{\mathrm{koff}_{p1}}{\overset{\mathrm{kon}_{p1}}{\rightleftharpoons}} C_1X_1N_{11}{}^D{-}C_2{}^EX_2N_{21}{}^D \tag{186}$$

$$C_1X_1N_{11}{}^D + C_1{}^EX_1N_{11}{}^D \underset{\mathrm{koff}_{p1}}{\overset{\mathrm{kon}_{p1}}{\rightleftharpoons}} C_1X_1N_{11}{}^D{-}C_1{}^EX_1N_{11}{}^D \tag{187}$$

$$C_1X_1N_{11}{}^D + C_1{}^EX_2N_{21}{}^D \underset{\mathrm{koff}_{p1}}{\overset{\mathrm{kon}_{p1}}{\rightleftharpoons}} C_1X_1N_{11}{}^D{-}C_1{}^EX_2N_{21}{}^D \tag{188}$$

$$C_1X_2N_{21}{}^D + C_2{}^EX_1N_{11}{}^D \underset{\mathrm{koff}_{p1}}{\overset{\mathrm{kon}_{p1}}{\rightleftharpoons}} C_1X_2N_{21}{}^D{-}C_2{}^EX_1N_{11}{}^D \tag{189}$$

$$C_1X_2N_{21}{}^D + C_2{}^EX_2N_{21}{}^D \underset{\mathrm{koff}_{p1}}{\overset{\mathrm{kon}_{p1}}{\rightleftharpoons}} C_1X_2N_{21}{}^D{-}C_2{}^EX_2N_{21}{}^D \tag{190}$$

$$C_1X_2N_{21}{}^D + C_1{}^EX_1N_{11}{}^D \underset{\mathrm{koff}_{p1}}{\overset{\mathrm{kon}_{p1}}{\rightleftharpoons}} C_1X_2N_{21}{}^D{-}C_1{}^EX_1N_{11}{}^D \tag{191}$$

$$C_1X_2N_{21}{}^D + C_1{}^EX_2N_{21}{}^D \underset{\mathrm{koff}_{p1}}{\overset{\mathrm{kon}_{p1}}{\rightleftharpoons}} C_1X_2N_{21}{}^D{-}C_1{}^EX_2N_{21}{}^D \tag{192}$$

$$C_2X_1N_{12}{}^D + C_2{}^EX_1N_{12}{}^D \underset{\mathrm{koff}_{p2}}{\overset{\mathrm{kon}_{p2}}{\rightleftharpoons}} C_2X_1N_{12}{}^D{-}C_2{}^EX_1N_{12}{}^D \tag{193}$$

$$C_2X_1N_{12}{}^D + C_2{}^EX_2N_{22}{}^D \underset{\mathrm{koff}_{p2}}{\overset{\mathrm{kon}_{p2}}{\rightleftharpoons}} C_2X_1N_{12}{}^D{-}C_2{}^EX_2N_{22}{}^D \tag{194}$$

$$C_2X_1N_{12}{}^D + C_1{}^EX_1N_{12}{}^D \underset{\mathrm{koff}_{p2}}{\overset{\mathrm{kon}_{p2}}{\rightleftharpoons}} C_2X_1N_{12}{}^D{-}C_1{}^EX_1N_{12}{}^D \tag{195}$$

$$C_2X_1N_{12}{}^D + C_1{}^EX_2N_{22}{}^D \underset{\mathrm{koff}_{p2}}{\overset{\mathrm{kon}_{p2}}{\rightleftharpoons}} C_2X_1N_{12}{}^D{-}C_1{}^EX_2N_{22}{}^D \tag{196}$$

$$C_2X_2N_{22}{}^D + C_2{}^EX_1N_{12}{}^D \underset{\mathrm{koff}_{p2}}{\overset{\mathrm{kon}_{p2}}{\rightleftharpoons}} C_2X_2N_{22}{}^D{-}C_2{}^EX_1N_{12}{}^D \tag{197}$$

$$C_2X_2N_{22}{}^D + C_2{}^EX_2N_{22}{}^D \underset{\mathrm{koff}_{p2}}{\overset{\mathrm{kon}_{p2}}{\rightleftharpoons}} C_2X_2N_{22}{}^D{-}C_2{}^EX_2N_{22}{}^D \tag{198}$$

$$C_2X_2N_{22}{}^D + C_1{}^EX_1N_{12}{}^D \underset{\mathrm{koff}_{p2}}{\overset{\mathrm{kon}_{p2}}{\rightleftharpoons}} C_2X_2N_{22}{}^D{-}C_1{}^EX_1N_{12}{}^D \tag{199}$$

$$C_2X_2N_{22}{}^D + C_1{}^EX_2N_{22}{}^D \underset{\mathrm{koff}_{p2}}{\overset{\mathrm{kon}_{p2}}{\rightleftharpoons}} C_2X_2N_{22}{}^D{-}C_1{}^EX_2N_{22}{}^D \tag{200}$$

$$C_1X_1N_{11}{}^{D}{-}C_2{}^{E}X_1N_{11}{}^{D} \xrightarrow{kcat_1} C_1X_1N_{11}{}^{D} + C_2{}^{E}X_1N_{11} \tag{201}$$

$$C_1X_1N_{11}{}^{D}{-}C_2{}^{E}X_2N_{21}{}^{D} \xrightarrow{kcat_1} C_1X_1N_{11}{}^{D} + C_2{}^{E}X_2N_{21} \tag{202}$$

$$C_1X_1N_{11}{}^{D}{-}C_1{}^{E}X_1N_{11}{}^{D} \xrightarrow{kcat_1} C_1X_1N_{11}{}^{D} + C_1{}^{E}X_1N_{11} \tag{203}$$

$$C_1X_1N_{11}{}^{D}{-}C_1{}^{E}X_2N_{21}{}^{D} \xrightarrow{kcat_1} C_1X_1N_{11}{}^{D} + C_1{}^{E}X_2N_{21} \tag{204}$$

$$C_1X_2N_{21}{}^{D}{-}C_2{}^{E}X_1N_{11}{}^{D} \xrightarrow{kcat_1} C_1X_2N_{21}{}^{D} + C_2{}^{E}X_1N_{11} \tag{205}$$

$$C_1X_2N_{21}{}^{D}{-}C_2{}^{E}X_2N_{21}{}^{D} \xrightarrow{kcat_1} C_1X_2N_{21}{}^{D} + C_2{}^{E}X_2N_{21} \tag{206}$$

$$C_1X_2N_{21}{}^{D}{-}C_1{}^{E}X_1N_{11}{}^{D} \xrightarrow{kcat_1} C_1X_2N_{21}{}^{D} + C_1{}^{E}X_1N_{11} \tag{207}$$

$$C_1X_2N_{21}{}^{D}{-}C_1{}^{E}X_2N_{21}{}^{D} \xrightarrow{kcat_1} C_1X_2N_{21}{}^{D} + C_1{}^{E}X_2N_{21} \tag{208}$$

$$C_2X_1N_{12}{}^{D}{-}C_2{}^{E}X_1N_{12}{}^{D} \xrightarrow{kcat_2} C_2X_1N_{12}{}^{D} + C_2{}^{E}X_1N_{12} \tag{209}$$

$$C_2X_1N_{12}{}^{D}{-}C_2{}^{E}X_2N_{22}{}^{D} \xrightarrow{kcat_2} C_2X_1N_{12}{}^{D} + C_2{}^{E}X_2N_{22} \tag{210}$$

$$C_2X_1N_{12}{}^{D}{-}C_1{}^{E}X_1N_{12}{}^{D} \xrightarrow{kcat_2} C_2X_1N_{12}{}^{D} + C_1{}^{E}X_1N_{12} \tag{211}$$

$$C_2X_1N_{12}{}^{D}{-}C_1{}^{E}X_2N_{22}{}^{D} \xrightarrow{kcat_2} C_2X_1N_{12}{}^{D} + C_1{}^{E}X_2N_{22} \tag{212}$$

$$C_2X_2N_{22}{}^{D}{-}C_2{}^{E}X_1N_{12}{}^{D} \xrightarrow{kcat_2} C_2X_2N_{22}{}^{D} + C_2{}^{E}X_1N_{12} \tag{213}$$

$$C_2X_2N_{22}{}^{D}{-}C_2{}^{E}X_2N_{22}{}^{D} \xrightarrow{kcat_2} C_2X_2N_{22}{}^{D} + C_2{}^{E}X_2N_{22} \tag{214}$$

$$C_2X_2N_{22}{}^{D}{-}C_1{}^{E}X_1N_{12}{}^{D} \xrightarrow{kcat_2} C_2X_2N_{22}{}^{D} + C_1{}^{E}X_1N_{12} \tag{215}$$

$$C_2X_2N_{22}{}^{D}{-}C_1{}^{E}X_2N_{22}{}^{D} \xrightarrow{kcat_2} C_2X_2N_{22}{}^{D} + C_1{}^{E}X_2N_{22} \tag{216}$$

$$C_1X_1N_{11} + C_2{}^{E}X_1N_{11}{}^{D} \xrightleftharpoons[koff_{p1}]{kon_{p1}} C_1X_1N_{11}{-}C_2{}^{E}X_1N_{11}{}^{D} \tag{217}$$

$$C_1X_1N_{11} + C_2{}^{E}X_2N_{21}{}^{D} \xrightleftharpoons[koff_{p1}]{kon_{p1}} C_1X_1N_{11}{-}C_2{}^{E}X_2N_{21}{}^{D} \tag{218}$$

$$C_1X_1N_{11} + C_1{}^{E}X_1N_{11}{}^{D} \xrightleftharpoons[koff_{p1}]{kon_{p1}} C_1X_1N_{11}{-}C_1{}^{E}X_1N_{11}{}^{D} \tag{219}$$

$$C_1X_1N_{11} + C_1{}^{E}X_2N_{21}{}^{D} \xrightleftharpoons[koff_{p1}]{kon_{p1}} C_1X_1N_{11}{-}C_1{}^{E}X_2N_{21}{}^{D} \tag{220}$$

$$C_1X_2N_{21} + C_2{}^{E}X_1N_{11}{}^{D} \xrightleftharpoons[koff_{p1}]{kon_{p1}} C_1X_2N_{21}{-}C_2{}^{E}X_1N_{11}{}^{D} \tag{221}$$

$$C_1X_2N_{21} + C_2{}^{E}X_2N_{21}{}^{D} \xrightleftharpoons[koff_{p1}]{kon_{p1}} C_1X_2N_{21}{-}C_2{}^{E}X_2N_{21}{}^{D} \tag{222}$$

$$C_1X_2N_{21} + C_1{}^{E}X_1N_{11}{}^{D} \xrightleftharpoons[koff_{p1}]{kon_{p1}} C_1X_2N_{21}{-}C_1{}^{E}X_1N_{11}{}^{D} \tag{223}$$

$$C_1X_2N_{21} + C_1{}^{E}X_2N_{21}{}^{D} \xrightleftharpoons[koff_{p1}]{kon_{p1}} C_1X_2N_{21}{-}C_1{}^{E}X_2N_{21}{}^{D} \tag{224}$$

$$C_2X_1N_{12} + C_2{}^{E}X_1N_{12}{}^{D} \xrightleftharpoons[koff_{p2}]{kon_{p2}} C_2X_1N_{12}{-}C_2{}^{E}X_1N_{12}{}^{D} \tag{225}$$

$$C_2X_1N_{12} + C_2{}^{E}X_2N_{22}{}^{D} \underset{\mathrm{koff}_{p2}}{\overset{\mathrm{kon}_{p2}}{\rightleftharpoons}} C_2X_1N_{12}{-}C_2{}^{E}X_2N_{22}{}^{D} \tag{226}$$

$$C_2X_1N_{12} + C_1{}^{E}X_1N_{12}{}^{D} \underset{\mathrm{koff}_{p2}}{\overset{\mathrm{kon}_{p2}}{\rightleftharpoons}} C_2X_1N_{12}{-}C_1{}^{E}X_1N_{12}{}^{D} \tag{227}$$

$$C_2X_1N_{12} + C_1{}^{E}X_2N_{22}{}^{D} \underset{\mathrm{koff}_{p2}}{\overset{\mathrm{kon}_{p2}}{\rightleftharpoons}} C_2X_1N_{12}{-}C_1{}^{E}X_2N_{22}{}^{D} \tag{228}$$

$$C_2X_2N_{22} + C_2{}^{E}X_1N_{12}{}^{D} \underset{\mathrm{koff}_{p2}}{\overset{\mathrm{kon}_{p2}}{\rightleftharpoons}} C_2X_2N_{22}{-}C_2{}^{E}X_1N_{12}{}^{D} \tag{229}$$

$$C_2X_2N_{22} + C_2{}^{E}X_2N_{22}{}^{D} \underset{\mathrm{koff}_{p2}}{\overset{\mathrm{kon}_{p2}}{\rightleftharpoons}} C_2X_2N_{22}{-}C_2{}^{E}X_2N_{22}{}^{D} \tag{230}$$

$$C_2X_2N_{22} + C_1{}^{E}X_1N_{12}{}^{D} \underset{\mathrm{koff}_{p2}}{\overset{\mathrm{kon}_{p2}}{\rightleftharpoons}} C_2X_2N_{22}{-}C_1{}^{E}X_1N_{12}{}^{D} \tag{231}$$

$$C_2X_2N_{22} + C_1{}^{E}X_2N_{22}{}^{D} \underset{\mathrm{koff}_{p2}}{\overset{\mathrm{kon}_{p2}}{\rightleftharpoons}} C_2X_2N_{22}{-}C_1{}^{E}X_2N_{22}{}^{D} \tag{232}$$

$$C_1X_1N_{11}{-}C_2{}^{E}X_1N_{11}{}^{D} \xrightarrow{\mathrm{kcat}_1} C_1X_1N_{11} + C_2{}^{E}X_1N_{11} \tag{233}$$

$$C_1X_1N_{11}{-}C_2{}^{E}X_2N_{21}{}^{D} \xrightarrow{\mathrm{kcat}_1} C_1X_1N_{11} + C_2{}^{E}X_2N_{21} \tag{234}$$

$$C_1X_1N_{11}{-}C_1{}^{E}X_1N_{11}{}^{D} \xrightarrow{\mathrm{kcat}_1} C_1X_1N_{11} + C_1{}^{E}X_1N_{11} \tag{235}$$

$$C_1X_1N_{11}{-}C_1{}^{E}X_2N_{21}{}^{D} \xrightarrow{\mathrm{kcat}_1} C_1X_1N_{11} + C_1{}^{E}X_2N_{21} \tag{236}$$

$$C_1X_2N_{21}{-}C_2{}^{E}X_1N_{11}{}^{D} \xrightarrow{\mathrm{kcat}_1} C_1X_2N_{21} + C_2{}^{E}X_1N_{11} \tag{237}$$

$$C_1X_2N_{21}{-}C_2{}^{E}X_2N_{21}{}^{D} \xrightarrow{\mathrm{kcat}_1} C_1X_2N_{21} + C_2{}^{E}X_2N_{21} \tag{238}$$

$$C_1X_2N_{21}{-}C_1{}^{E}X_1N_{11}{}^{D} \xrightarrow{\mathrm{kcat}_1} C_1X_2N_{21} + C_1{}^{E}X_1N_{11} \tag{239}$$

$$C_1X_2N_{21}{-}C_1{}^{E}X_2N_{21}{}^{D} \xrightarrow{\mathrm{kcat}_1} C_1X_2N_{21} + C_1{}^{E}X_2N_{21} \tag{240}$$

$$C_2X_1N_{12}{-}C_2{}^{E}X_1N_{12}{}^{D} \xrightarrow{\mathrm{kcat}_2} C_2X_1N_{12} + C_2{}^{E}X_1N_{12} \tag{241}$$

$$C_2X_1N_{12}{-}C_2{}^{E}X_2N_{22}{}^{D} \xrightarrow{\mathrm{kcat}_2} C_2X_1N_{12} + C_2{}^{E}X_2N_{22} \tag{242}$$

$$C_2X_1N_{12}{-}C_1{}^{E}X_1N_{12}{}^{D} \xrightarrow{\mathrm{kcat}_2} C_2X_1N_{12} + C_1{}^{E}X_1N_{12} \tag{243}$$

$$C_2X_1N_{12}{-}C_1{}^{E}X_2N_{22}{}^{D} \xrightarrow{\mathrm{kcat}_2} C_2X_1N_{12} + C_1{}^{E}X_2N_{22} \tag{244}$$

$$C_2X_2N_{22}{-}C_2{}^{E}X_1N_{12}{}^{D} \xrightarrow{\mathrm{kcat}_2} C_2X_2N_{22} + C_2{}^{E}X_1N_{12} \tag{245}$$

$$C_2X_2N_{22}{-}C_2{}^{E}X_2N_{22}{}^{D} \xrightarrow{\mathrm{kcat}_2} C_2X_2N_{22} + C_2{}^{E}X_2N_{22} \tag{246}$$

$$C_2X_2N_{22}{-}C_1{}^{E}X_1N_{12}{}^{D} \xrightarrow{\mathrm{kcat}_2} C_2X_2N_{22} + C_1{}^{E}X_1N_{12} \tag{247}$$

$$C_2X_2N_{22}{-}C_1{}^{E}X_2N_{22}{}^{D} \xrightarrow{\mathrm{kcat}_2} C_2X_2N_{22} + C_1{}^{E}X_2N_{22} \tag{248}$$

$$\varnothing \xrightarrow{\mathrm{syn}_{X1}/(\mathrm{avgd}\cdot\mathrm{cell})} X_1 \tag{249}$$

$$\varnothing \xrightarrow{\mathrm{syn}_{X2}/(\mathrm{avgd}\cdot\mathrm{cell})} X_2 \tag{250}$$

$$\varnothing \xrightarrow{\mathrm{syn}_{\mathrm{N11}}\,/(\mathrm{avgd}\cdot\mathrm{cell})} \mathrm{N_{11}}^{\mathrm{D}} \tag{251}$$

$$\varnothing \xrightarrow{\mathrm{syn}_{\mathrm{N12}}\,/(\mathrm{avgd}\cdot\mathrm{cell})} \mathrm{N_{12}}^{\mathrm{D}} \tag{252}$$

$$\varnothing \xrightarrow{\mathrm{syn}_{\mathrm{N21}}\,/(\mathrm{avgd}\cdot\mathrm{cell})} \mathrm{N_{21}}^{\mathrm{D}} \tag{253}$$

$$\varnothing \xrightarrow{\mathrm{syn}_{\mathrm{N22}}\,/(\mathrm{avgd}\cdot\mathrm{cell})} \mathrm{N_{22}}^{\mathrm{D}} \tag{254}$$

$$\varnothing \xrightarrow{\mathrm{syn{-}C_1}\,/(\mathrm{avgd}\cdot\mathrm{cell})} \mathrm{C_1} \tag{255}$$

$$\varnothing \xrightarrow{\mathrm{syn{-}C_2}\,/(\mathrm{avgd}\cdot\mathrm{cell})} \mathrm{C_2} \tag{256}$$

$$\mathrm{X_1N_{11}}^{\mathrm{D}} \xrightarrow{\mathrm{deg}_{\mathrm{DHFR}}} \varnothing \tag{257}$$

$$\mathrm{X_1N_{12}}^{\mathrm{D}} \xrightarrow{\mathrm{deg}_{\mathrm{DHFR}}} \varnothing \tag{258}$$

$$\mathrm{X_2N_{21}}^{\mathrm{D}} \xrightarrow{\mathrm{deg}_{\mathrm{DHFR}}} \varnothing \tag{259}$$

$$\mathrm{X_2N_{22}}^{\mathrm{D}} \xrightarrow{\mathrm{deg}_{\mathrm{DHFR}}} \varnothing \tag{260}$$

$$\mathrm{C_1X_1N_{11}}^{\mathrm{D}} \xrightarrow{\mathrm{deg}_{\mathrm{DHFR}}} \varnothing \tag{261}$$

$$\mathrm{C_1X_1N_{12}}^{\mathrm{D}} \xrightarrow{\mathrm{deg}_{\mathrm{DHFR}}} \varnothing \tag{262}$$

$$\mathrm{C_1X_2N_{21}}^{\mathrm{D}} \xrightarrow{\mathrm{deg}_{\mathrm{DHFR}}} \varnothing \tag{263}$$

$$\mathrm{C_1X_2N_{22}}^{\mathrm{D}} \xrightarrow{\mathrm{deg}_{\mathrm{DHFR}}} \varnothing \tag{264}$$

$$\mathrm{C_2X_1N_{11}}^{\mathrm{D}} \xrightarrow{\mathrm{deg}_{\mathrm{DHFR}}} \varnothing \tag{265}$$

$$\mathrm{C_2X_1N_{12}}^{\mathrm{D}} \xrightarrow{\mathrm{deg}_{\mathrm{DHFR}}} \varnothing \tag{266}$$

$$\mathrm{C_2X_2N_{21}}^{\mathrm{D}} \xrightarrow{\mathrm{deg}_{\mathrm{DHFR}}} \varnothing \tag{267}$$

$$\mathrm{C_2X_2N_{22}}^{\mathrm{D}} \xrightarrow{\mathrm{deg}_{\mathrm{DHFR}}} \varnothing \tag{268}$$

$$\mathrm{N_{11}} \xrightarrow{\mathrm{deg}_{\mathrm{reg}}} \varnothing \tag{269}$$

$$\mathrm{N_{21}} \xrightarrow{\mathrm{deg}_{\mathrm{reg}}} \varnothing \tag{270}$$

$$\mathrm{N_{12}} \xrightarrow{\mathrm{deg}_{\mathrm{reg}}} \varnothing \tag{271}$$

$$\mathrm{N_{22}} \xrightarrow{\mathrm{deg}_{\mathrm{reg}}} \varnothing \tag{272}$$

$$\mathrm{X_1N_{11}} \xrightarrow{\mathrm{deg}_{\mathrm{reg}}} \varnothing \tag{273}$$

$$\mathrm{X_2N_{21}} \xrightarrow{\mathrm{deg}_{\mathrm{reg}}} \varnothing \tag{274}$$

$$\mathrm{X_1N_{12}} \xrightarrow{\mathrm{deg}_{\mathrm{reg}}} \varnothing \tag{275}$$

$$\mathrm{X_2N_{22}} \xrightarrow{\deg_{\mathrm{reg}}} \varnothing \tag{276}$$

$$\mathrm{C_1X_1N_{11}} \xrightarrow{\deg_{\mathrm{reg}}} \varnothing \tag{277}$$

$$\mathrm{C_1X_2N_{21}} \xrightarrow{\deg_{\mathrm{reg}}} \varnothing \tag{278}$$

$$\mathrm{C_2X_1N_{11}} \xrightarrow{\deg_{\mathrm{reg}}} \varnothing \tag{279}$$

$$\mathrm{C_2X_2N_{21}} \xrightarrow{\deg_{\mathrm{reg}}} \varnothing \tag{280}$$

$$\mathrm{C_1X_1N_{12}} \xrightarrow{\deg_{\mathrm{reg}}} \varnothing \tag{281}$$

$$\mathrm{C_1X_2N_{22}} \xrightarrow{\deg_{\mathrm{reg}}} \varnothing \tag{282}$$

$$\mathrm{C_2X_1N_{12}} \xrightarrow{\deg_{\mathrm{reg}}} \varnothing \tag{283}$$

$$\mathrm{C_2X_2N_{22}} \xrightarrow{\deg_{\mathrm{reg}}} \varnothing \tag{284}$$

$$\mathrm{C_2}^{\mathrm{E}} \xrightarrow{\deg_{\mathrm{reg}}} \varnothing \tag{285}$$

$$\mathrm{C_1}^{\mathrm{E}} \xrightarrow{\deg_{\mathrm{reg}}} \varnothing \tag{286}$$

$$\mathrm{C_2}^{\mathrm{E}}\mathrm{X_1N_{11}}^{\mathrm{D}} \xrightarrow{\deg_{\mathrm{DHFR}}} \varnothing \tag{287}$$

$$\mathrm{C_2}^{\mathrm{E}}\mathrm{X_1N_{12}}^{\mathrm{D}} \xrightarrow{\deg_{\mathrm{DHFR}}} \varnothing \tag{288}$$

$$\mathrm{C_2}^{\mathrm{E}}\mathrm{X_2N_{21}}^{\mathrm{D}} \xrightarrow{\deg_{\mathrm{DHFR}}} \varnothing \tag{289}$$

$$\mathrm{C_2}^{\mathrm{E}}\mathrm{X_2N_{22}}^{\mathrm{D}} \xrightarrow{\deg_{\mathrm{DHFR}}} \varnothing \tag{290}$$

$$\mathrm{C_1}^{\mathrm{E}}\mathrm{X_1N_{11}}^{\mathrm{D}} \xrightarrow{\deg_{\mathrm{DHFR}}} \varnothing \tag{291}$$

$$\mathrm{C_1}^{\mathrm{E}}\mathrm{X_1N_{12}}^{\mathrm{D}} \xrightarrow{\deg_{\mathrm{DHFR}}} \varnothing \tag{292}$$

$$\mathrm{C_1}^{\mathrm{E}}\mathrm{X_2N_{21}}^{\mathrm{D}} \xrightarrow{\deg_{\mathrm{DHFR}}} \varnothing \tag{293}$$

$$\mathrm{C_1}^{\mathrm{E}}\mathrm{X_2N_{22}}^{\mathrm{D}} \xrightarrow{\deg_{\mathrm{DHFR}}} \varnothing \tag{294}$$

$$\mathrm{C_2}^{\mathrm{E}}\mathrm{X_1N_{11}} \xrightarrow{\deg_{\mathrm{reg}}} \varnothing \tag{295}$$

$$\mathrm{C_2}^{\mathrm{E}}\mathrm{X_2N_{21}} \xrightarrow{\deg_{\mathrm{reg}}} \varnothing \tag{296}$$

$$\mathrm{C_1}^{\mathrm{E}}\mathrm{X_1N_{11}} \xrightarrow{\deg_{\mathrm{reg}}} \varnothing \tag{297}$$

$$\mathrm{C_1}^{\mathrm{E}}\mathrm{X_2N_{21}} \xrightarrow{\deg_{\mathrm{reg}}} \varnothing \tag{298}$$

$$\mathrm{C_2}^{\mathrm{E}}\mathrm{X_1N_{12}} \xrightarrow{\deg_{\mathrm{reg}}} \varnothing \tag{299}$$

$$\mathrm{C_2}^{\mathrm{E}}\mathrm{X_2N_{22}} \xrightarrow{\deg_{\mathrm{reg}}} \varnothing \tag{300}$$

$$\mathrm{C_1}^{\mathrm{E}}\mathrm{X_1N_{12}} \xrightarrow{\deg_{\mathrm{reg}}} \varnothing \tag{301}$$

$$\mathrm{C_1}^{\mathrm{E}}\mathrm{X_2N_{22}} \xrightarrow{\deg_{\mathrm{reg}}} \varnothing \tag{302}$$

$$\mathrm{X_1} \xrightarrow{\deg_{\mathrm{reg}}} \varnothing \tag{303}$$

$$\mathrm{X_2} \xrightarrow{\deg_{\mathrm{reg}}} \varnothing \tag{304}$$

$$\mathrm{N_{11}}^{\mathrm{D}} \xrightarrow{\deg_{\mathrm{DHFR}}} \varnothing \tag{305}$$

$$\mathrm{N_{12}}^{\mathrm{D}} \xrightarrow{\deg_{\mathrm{DHFR}}} \varnothing \tag{306}$$

$$\mathrm{N_{21}}^{\mathrm{D}} \xrightarrow{\deg_{\mathrm{DHFR}}} \varnothing \tag{307}$$

$$\mathrm{N_{22}}^{\mathrm{D}} \xrightarrow{\deg_{\mathrm{DHFR}}} \varnothing \tag{308}$$

$$\mathrm{C_1} \xrightarrow{\deg_{\mathrm{reg}}} \varnothing \tag{309}$$

$$\mathrm{C_2} \xrightarrow{\deg_{\mathrm{reg}}} \varnothing \tag{310}$$

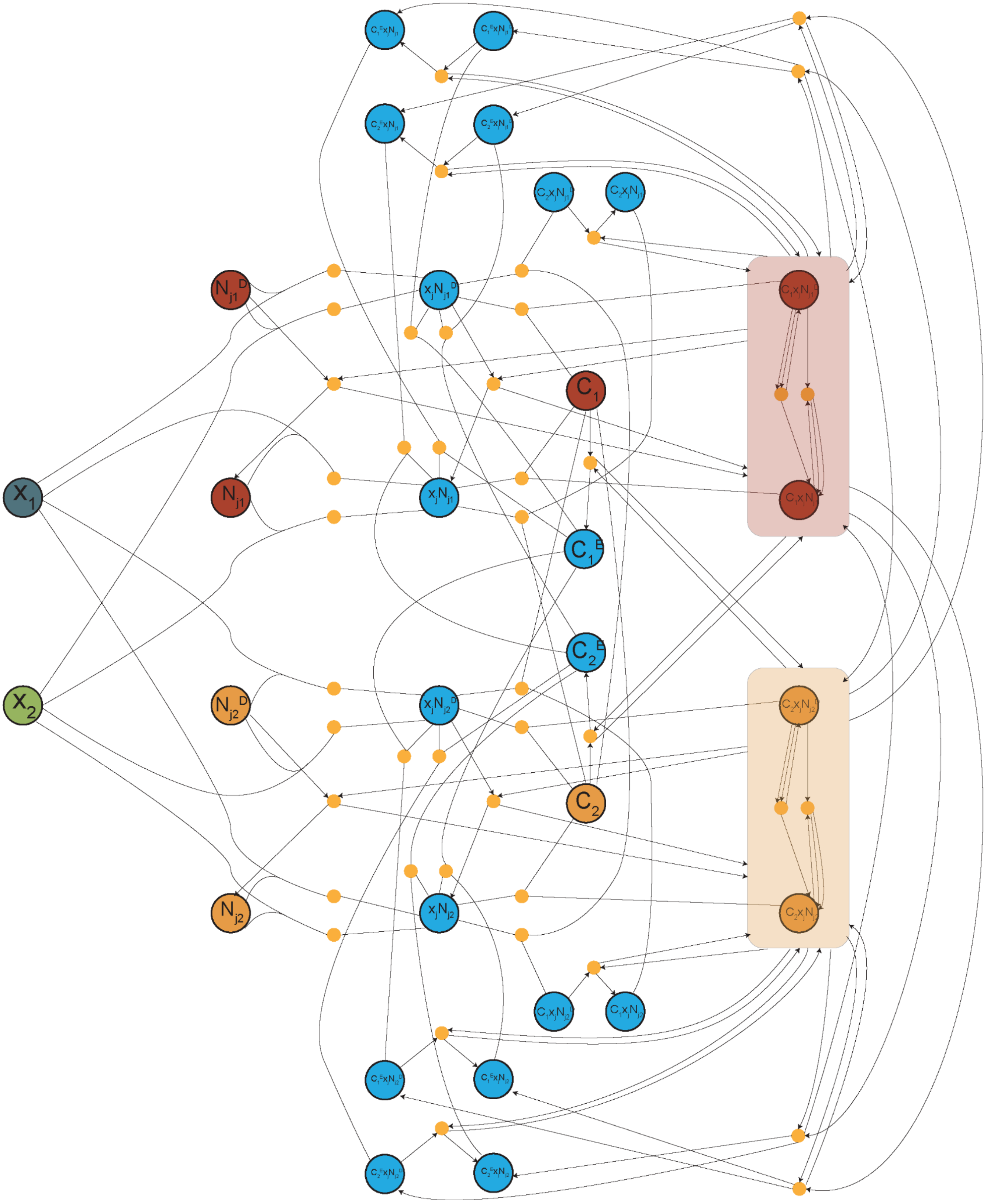
X1
X2
Nj1D
Nj1
Nj2D
Nj2
xjNj1D
xjNj1
xjNj2D
xjNj2
C1
C1E
C2E
C2

Figure S1. A chemical reaction network of the 2-input comparator. Protein species are grouped whenever possible to improve clarity. In these cases, the index j can be either 1 or 2. Circles represent a single protein or a group of protein species. Inputs and node proteins use the same color scheme as that in the main text, while intermediate and waste products are colored in blue. Orange dots represent a single or a group of chemical reactions. Lines indicate reversible reactions, lines with arrows indicate irreversible reactions. Protein synthesis, degradation, and enzyme-substrate complex formation are omitted.

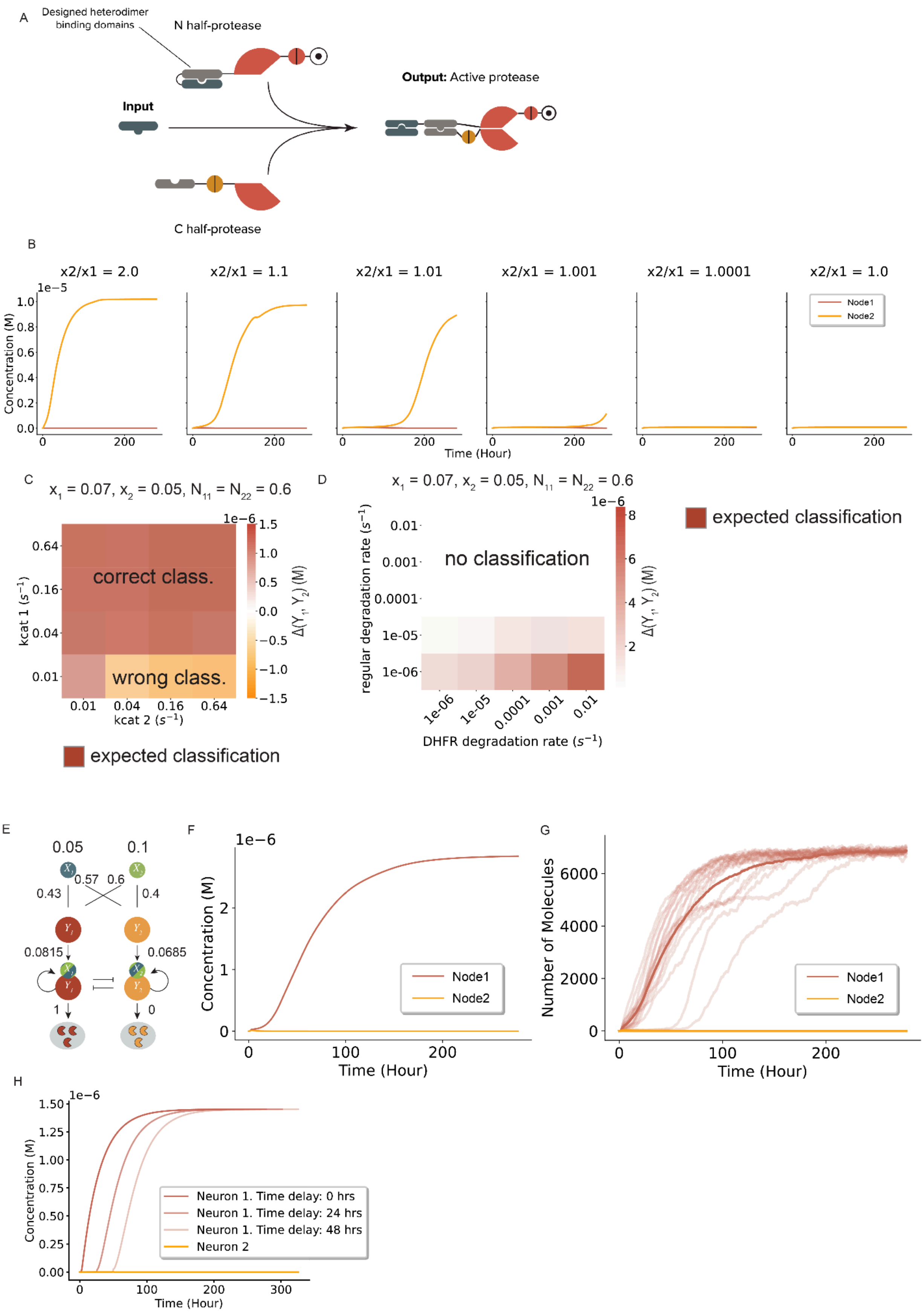

A
Designed heterodimer binding domains
N half-protease
Input
Output: Active protease
C half-protease
B
x2/x1 = 2.0
x2/x1 = 1.1
x2/x1 = 1.01
x2/x1 = 1.001
x2/x1 = 1.0001
x2/x1 = 1.0
Concentration (M)
Time (Hour)
Node1
Node2
C
x1 = 0.07, x2 = 0.05, N11 = N22 = 0.6
correct class.
wrong class.
kcat 1 (s−1)
kcat 2 (s−1)
Δ(Y1, Y2) (M)
expected classification
D
x1 = 0.07, x2 = 0.05, N11 = N22 = 0.6
no classification
regular degradation rate (s−1)
DHFR degradation rate (s−1)
Δ(Y1, Y2) (M)
expected classification
E
F
Concentration (M)
Time (Hour)
Node1
Node2
G
Number of Molecules
Time (Hour)
Node1
Node2
H
Concentration (M)
Time (Hour)
Neuron 1. Time delay: 0 hrs
Neuron 1. Time delay: 24 hrs
Neuron 1. Time delay: 48 hrs
Neuron 2

Figure S2. Simulations of the winner-take-all neural network. (A) The node, consisting of N and C half-proteases, is activated when the input protein is present. This activation mechanism makes use of a previously characterized cooperative binding scheme, which favors the trimeric complex over partial dimeric complexes. (B) Deterministic simulation of the 2-input comparator with varying input levels. The circuit takes longer to classify inputs that are increasingly similar to each other, and can distinguish inputs with concentration differences of as little as 10% within a reasonable timeframe. (C) For a 2-input comparator, scanning the k_{cat} parameter values of the two proteases reveal that the circuit can correctly classify relative input levels as long as both proteases have a k_{cat} value of at least 0.04/s. (D) For a 2-input comparator, bigger differences between the basal degradation rate (y-axis) and degron-based degradation rates (x-axis) result in more accurate classification (colorbar). (E) A fully connected 2-input neural network generates similar simulated dynamics as the comparator in both deterministic (F) and stochastic (G) simulations. (H) The perceptein circuit reaches the same steady state despite delays in inputs. Synthesis rates for input proteins were kept at 0 until the indicated delay time, after which the synthesis rates for X_1 and X_2 were switched to 0.07/s and 0.05/s, respectively.

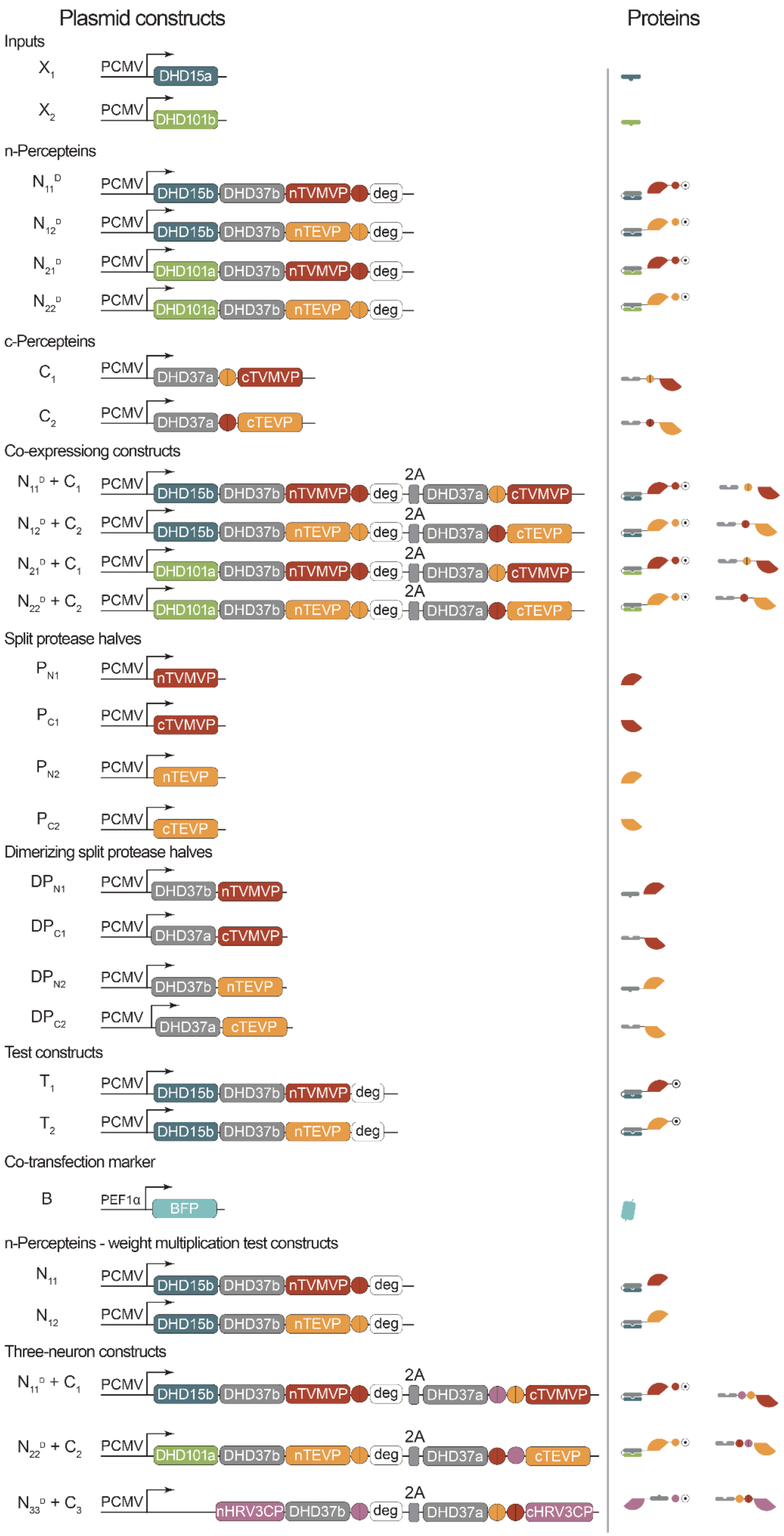

Plasmid constructs
Proteins
Inputs
X1
PCMV
DHD15a
X2
DHD101b
n-Percepteins
N11D
DHD15b
DHD37b
nTVMVP
deg
N12D
nTEVP
N21D
DHD101a
N22D
c-Percepteins
C1
DHD37a
cTVMVP
C2
cTEVP
Co-expressiong constructs
N11D + C1
2A
N12D + C2
N21D + C1
N22D + C2
Split protease halves
PN1
PC1
PN2
PC2
Dimerizing split protease halves
DPN1
DPC1
DPN2
DPC2
Test constructs
T1
T2
Co-transfection marker
B
PEF1α
BFP
n-Percepteins - weight multiplication test constructs
N11
N12
Three-neuron constructs
N11D + C1
N22D + C2
N33D + C3
nHRV3CP
cHRV3CP

Figure S3. Complete list of plasmids and the encoded protein constructs. PCMV, human cytomegalovirus promoter. PEF1α, human elongation factor-1 α promoter. PGK, 3-phosphoglycerate kinase promoter. Deg, DHFR degron. BFP, blue fluorescent protein. Schematics of the resulting constructs are shown on the right. Orange and yellow circles denote cleavage sites for corresponding proteases.

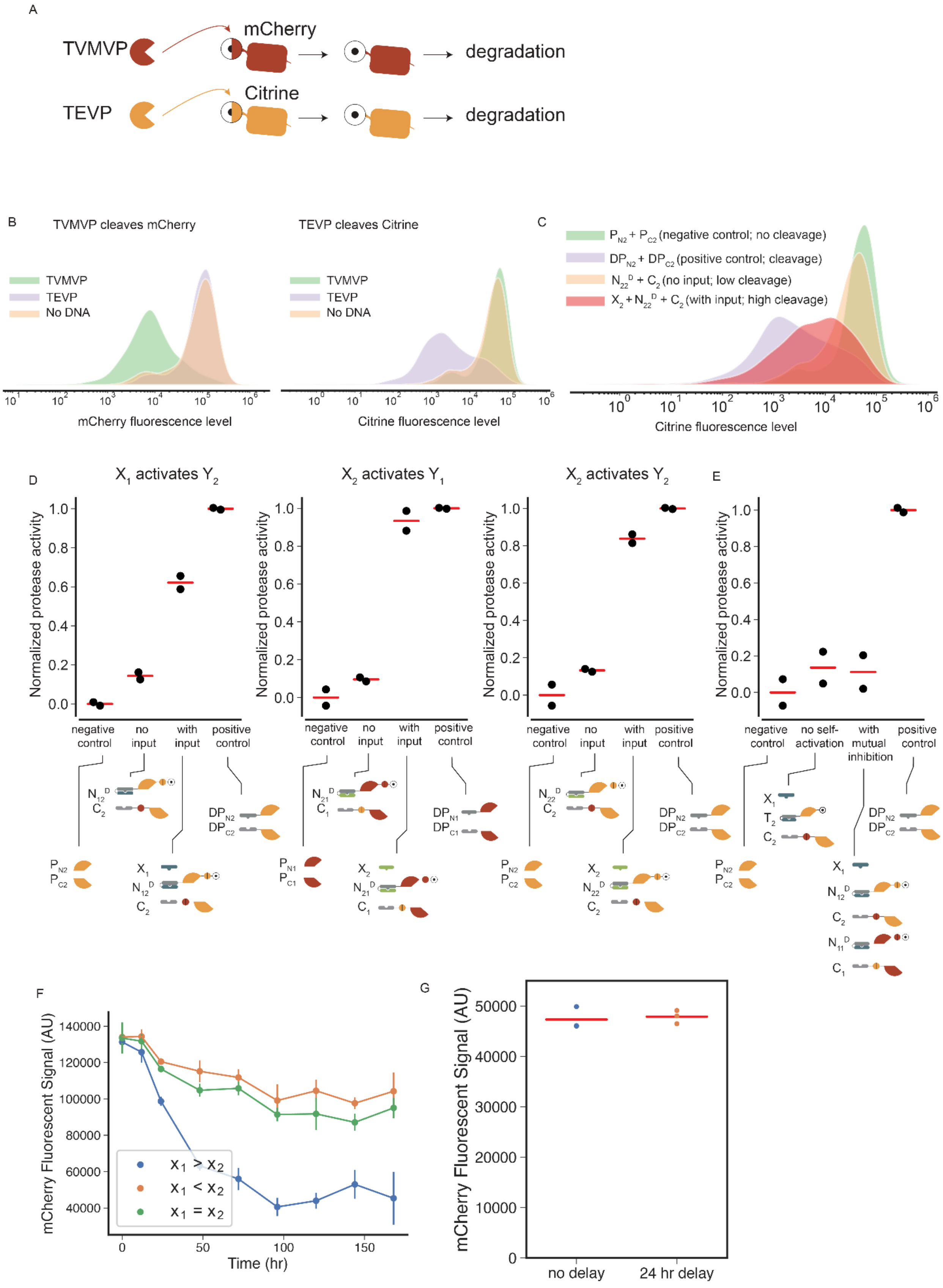
A
TVMVP
mCherry
degradation
TEVP
Citrine
degradation
B
TVMVP cleaves mCherry
TVMVP
TEVP
No DNA
mCherry fluorescence level
TEVP cleaves Citrine
TVMVP
TEVP
No DNA
Citrine fluorescence level
C
P_{N2} + P_{C2} (negative control; no cleavage)
DP_{N2} + DP_{C2} (positive control; cleavage)
N_{22}^D + C_2 (no input; low cleavage)
X_2 + N_{22}^D + C_2 (with input; high cleavage)
Citrine fluorescence level
D
X_1 activates Y_2
X_2 activates Y_1
X_2 activates Y_2
Normalized protease activity
negative control
no input
with input
positive control
E
negative control
no self-activation
with mutual inhibition
positive control
F
mCherry Fluorescent Signal (AU)
Time (hr)
$x_1 > x_2$
$x_1 < x_2$
$x_1 = x_2$
G
mCherry Fluorescent Signal (AU)
no delay
24 hr delay

Figure S4. Experimental validation of the winner-take-all neural network. (A) To read out protease activities, the reporter cell line (Figure 4B) expresses mCherry and Citrine fluorescent proteins with N-end degrons that can be revealed upon protease cleavage by TVMVP and TEVP, respectively. Schematic shows how cleavage can expose a degron (half-circle), to cause target protein degradation. (B) Flow cytometry histograms reveal that TVMVP and TEVP exclusively destabilize mCherry and Citrine, respectively. (C) A representative flow cytometry histogram showing the activation of Node 2 by the $N_{22}D$ protein and its input X_2. (D) Activation of other nodes by their corresponding input proteins. (E) The node protein $N_{12}D$ undergoes self-activation, and its activities can be inhibited by the opposing node protein $N_{11}D$. (F) Time course of the perceptein classifier experiment. Cells were transfected with plasmids encoding the node constructs, along with varying amounts of X_1 or X_2 input plasmids in the following combinations: (X_1 = 160 ng, X_2 = 0 ng), (X_1 = 0 ng, X_2 = 160 ng), (X_1 = 160 ng, X_2 = 160 ng). Fluorescent signals from mCherry were monitored over time. Data represents three biological repeats. (G) Input plasmids (X_1 = 320 ng, X_2 = 0 ng) were transfected into HEK1012 cells either simultaneously with the node plasmids (left), or 24 hours after transfecting the node plasmids (right). Fluorescent signals remained largely unchanged, indicating that the circuit works equally well when there is a delay in inputs.

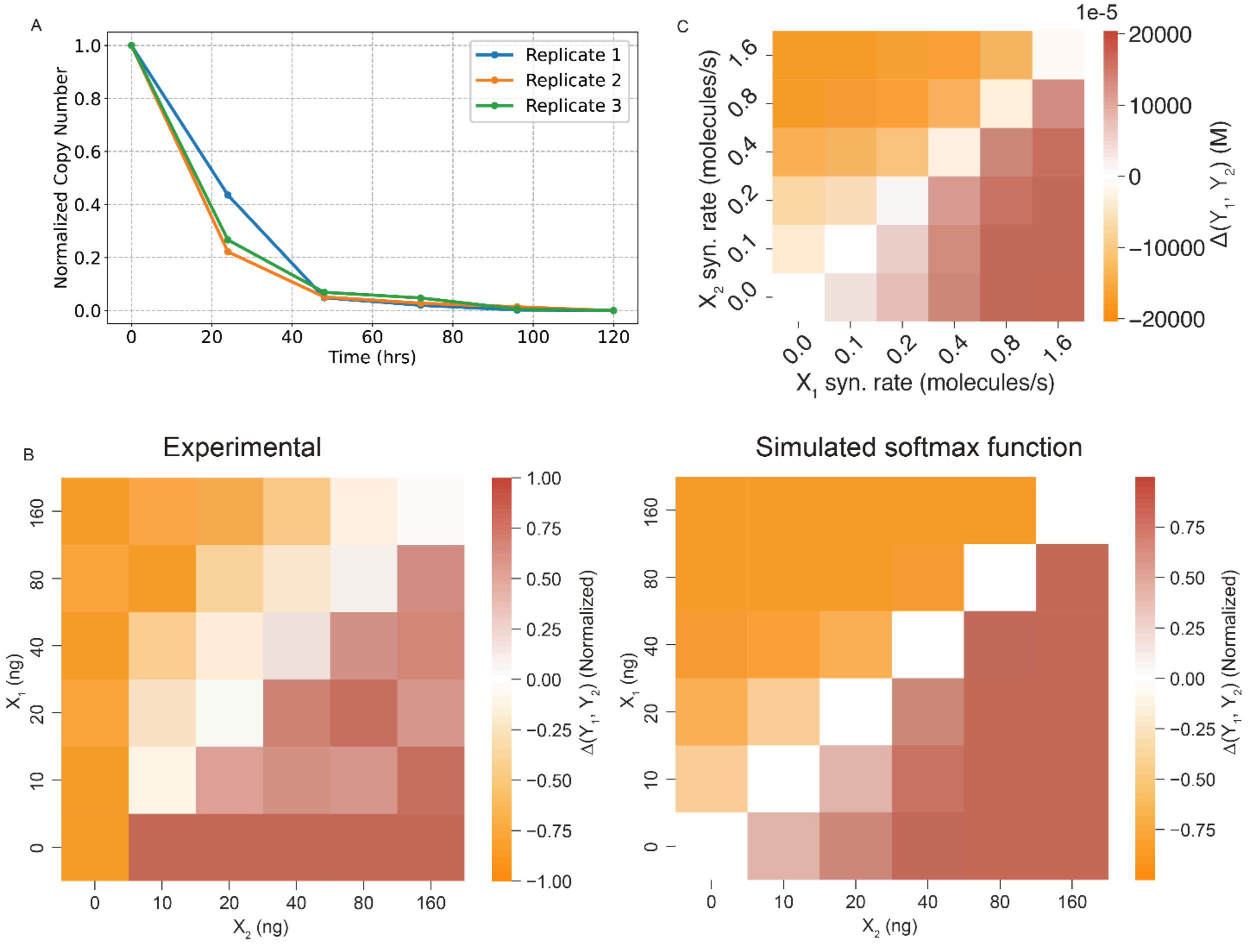

A
Normalized Copy Number
Time (hrs)
Replicate 1
Replicate 2
Replicate 3
C
1e-5
X_2 syn. rate (molecules/s)
X_1 syn. rate (molecules/s)
$\Delta(Y_1, Y_2)$ (M)
B
Experimental
Simulated softmax function
X_1 (ng)
X_2 (ng)
$\Delta(Y_1, Y_2)$ (Normalized)

Figure S5. (A) mRNA copy numbers in HEK293 T-REx cells 24-hour post transfection (t=0) were monitored over time with RT-qPCR in three biological repeats. (B) Data from the unbiased comparator (left) was normalized and fit to a softmax function in the form of $softmax(x_i) = e^{\beta x_i} / \sum_{j=1}^{K} e^{\beta x_j} \, for \; i = 1, ..., K$. The free parameter β, which determines the sharpness of the softmax function's output probabilities, that gave the best fit (right) was found to be 0.1± 0.065. (C) Stochastic simulation of the comparator circuit reveals that intrinsic noise causes analog, rather than all-or-none classification behavior. Each data point on the heat map is the average of 20 independent simulations.

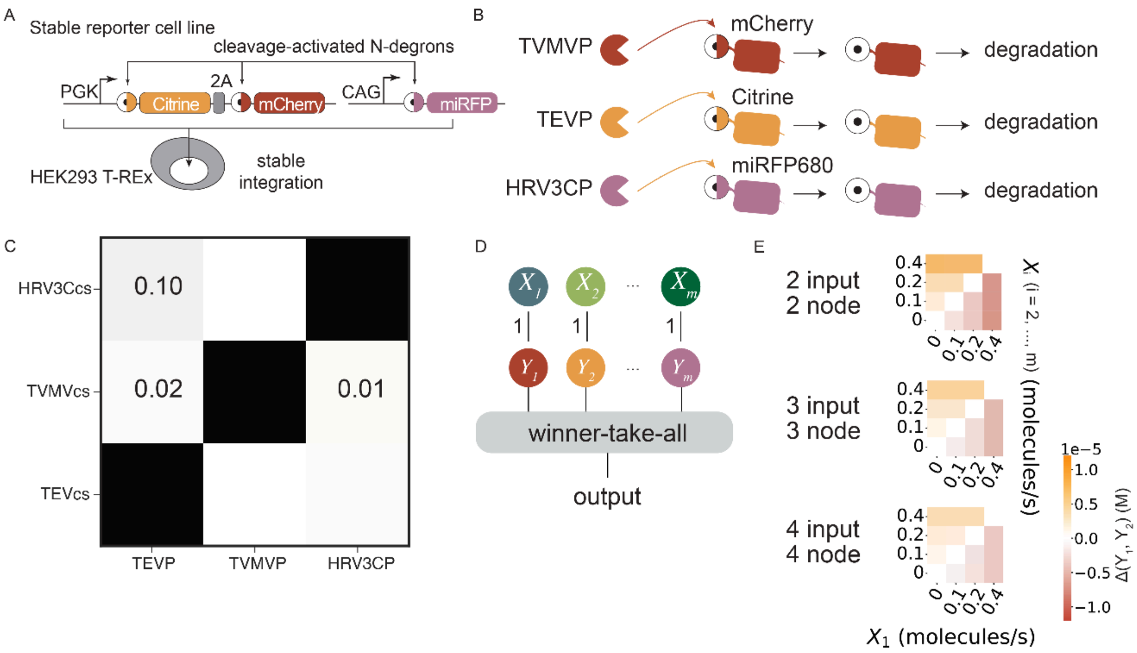
A
Stable reporter cell line
cleavage-activated N-degrons
PGK
2A
Citrine
mCherry
CAG
miRFP
HEK293 T-REx
stable
integration
B
TVMVP
mCherry
degradation
TEVP
Citrine
degradation
HRV3CP
miRFP680
degradation
C
HRV3Ccs
TVMVcs
TEVcs
0.10
0.02
0.01
TEVP
TVMVP
HRV3CP
D
winner-take-all
output
E
2 input
2 node
3 input
3 node
4 input
4 node
X_i (i = 2, ..., m) (molecules/s)
X_1 (molecules/s)
1e−5
Δ(Y₁, Y₂) (M)

Figure S6. (A) The stable reporter cell line HEK1013 constitutively co-expresses Citrine, mCherry, and miRFP680 fluorescent proteins that can be cleaved at the N-terminus by TEVP, TVMVP, and HRV3CP, respectively, to reveal N-terminal degrons that destabilize the fluorescent proteins. PGK, 3-phosphoglycerate kinase promoter. CAG, cytomegalovirus early enhancer/chicken β-actin promoter. (B) To read out protease activities, the reporter cell line HEK1013 in (A) expresses mCherry, Citrine, and miRFP680 fluorescent proteins with N-end degrons that can be revealed upon protease cleavage by TVMVP, TEVP, and HRV3CP, respectively. Schematic shows how cleavage can expose a degron (half-circle), to cause target protein degradation. (C) Relative fluorescent signal reduction in HEK1013 cells when plasmids encoding the three proteases were individually transfected. cs, protease cut site. Data points are averages of three biological repeats and are normalized independently in each row. Black and white cells represent complete and no cleavage, respectively. (D) An m-input, m-node comparator circuit. (E) Classification abilities of comparators that take 2, 3, and 4 inputs. As the size of the comparator increases, the ability to compare relative input levels is retained, while dynamic range is reduced due to the increased total number of substrates for each protease.

Table S1. Reaction rates used in simulations.

Rates	Description	Value	Reference
kon_1	on rate for the first step of cooperative DHD binding	10^5 s^{-1} M^{-1}	(*32*)
$koff_1$	off rate for the first step of cooperative DHD binding	100 s^{-1}	(*32*)
kon_2	on rate for the second step of cooperative DHD binding	10^5 s^{-1} M^{-1}	(*32*)
$koff_2$	off rate for the second step of cooperative DHD binding	10^{-4} s^{-1}	(*32*)
kon_p	on rate between proteases and substrates	10^5 s^{-1} M^{-1}	estimated
$koff_p$	off rate between proteases and substrates	10^{-4} s^{-1}	estimated
deg_{reg}	regular protein degradation rate	10^{-5} s^{-1}	(*49*)
deg_{DHFR}	degradation rate for DHFR-tagged proteins	10^{-3} s^{-1} - 10^{-2} s^{-1}	(*50*)
k_{cat}	protease turnover number	0.16 s^{-1}	(*51*)
V_{cell}	mammalian cell volume	$4*10^{-15}$ L	(*52*)
k_{syn}	protein synthesis rate	0.1-10 s^{-1}	estimated

Table S2. Proteins used in this study.

Name used in modeling and simulation	Protein domains ("-" denotes a flexible linker)	Symbol	Sequence
X_1	DHD15A	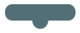	TREELLRENIELAKE HIEIMREILELLQKM EELLEKARGADEDV AKTIKELLRRLKEIIE RNQRIAKEHEYIARE RSS

X_2	DHD101B	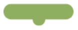	GSDAYDLDRIVKEH<br>RRLVEEQRELVEEL<br>EKLVRRQEDHRVDK<br>KESHEILERLERIIRR<br>STRILTELEKLTDEF<br>ERRTR
N_{11}^{D}	DHD15B-DHD37B-nTVMVP-TVMVcs-DHFR	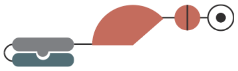	GTERKLLERSRRLQ<br>EESKRLLDEMAEIM<br>RRIKKLLKKARGAD<br>EKVLDELRKIIERIRE<br>LLDRSRKIHERSEEI<br>AYKEEGSEGSGSE<br>GSGSDDKELDKLLD<br>TLEKILQTATKIIDDA<br>NKLLEKLRRSERKD<br>PKVVETYVELLKRH<br>EKAVKELLEIAKTHA<br>KKVEGSEGSGSEG<br>SSKALLKGVRDFNPI<br>SACVCLLENSSDGH<br>SERLFGIGFGPYIIA<br>NQHLFRRNNGELTI<br>KTMHGEFKVKNST<br>QLQMKPVEGRDIIVI<br>KMAKDFPPFPQKLK<br>FRQPTIKDRVCMVS<br>TNFQQSGGGSSET<br>VRFQSGSGSISLIAA<br>LAVDYVIGMENAMP<br>WNLPADLAWFKRN<br>TLNKPVIMGRHTWE<br>SIGRPLPGRKNIILS<br>SQPSTDDRVTWVK<br>SVDEAIAACGDVPEI<br>MVIGGGRVIEQFLP<br>KAQKLYLTHIDAEVE<br>GDTHFPDYEPDDW<br>ESVFSEFHDADAQN<br>SHSYCFEILERR
N_{12}^{D}	DHD15B-DHD37B-nTEVP-TEVcs-DHFR	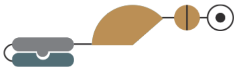	GTERKLLERSRRLQ<br>EESKRLLDEMAEIM<br>RRIKKLLKKARGAD<br>EKVLDELRKIIERIRE<br>LLDRSRKIHERSEEI<br>AYKEEGSEGSGSE<br>GSGSDDKELDKLLD<br>TLEKILQTATKIIDDA<br>NKLLEKLRRSERKD

			PKVVETYVELLKRH<br>EKAVKELLEIAKTHA<br>KKVEGSEGSGSEG<br>SGESLFKGPRDYNP<br>ISSTICHLTNESDGH<br>TTSLYGIGFGPFIITN<br>KHLFRRNNGTLLVQ<br>SLHGVFKVKNTTTL<br>QQHLIDGRDMIIIRM<br>PKDFPPFPQKLKFR<br>EPQREERICLVTTNF<br>QTGGGSSENLYFQ<br>SGSGSISLIAALAVD<br>YVIGMENAMPWNL<br>PADLAWFKRNTLNK<br>PVIMGRHTWESIGR<br>PLPGRKNIILSSQPS<br>TDDRVTWVKSVDE<br>AIAACGDVPEIMVIG<br>GGRVIEQFLPKAQK<br>LYLTHIDAEVEGDTH<br>FPDYEPDDWESVF<br>SEFHDADAQNSHS<br>YCFEILERR
N_{21}^{D}	DHD101A-DHD37B-nTVMVP-TVMVcs-DHFR	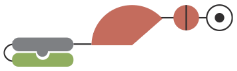	DEKDYHRRLIEHLE<br>DLVRRHEELIKRQK<br>KVVEELERRGLDER<br>LRRVVDRFRRSSER<br>WEEVIERFRQVVDK<br>LRKSVEGSEGSGSE<br>GSGSDDKELDKLLD<br>TLEKILQTATKIIDDA<br>NKLLEKLRRSERKD<br>PKVVETYVELLKRH<br>EKAVKELLEIAKTHA<br>KKVEGSEGSGSEG<br>SSKALLKGVRDFNPI<br>SACVCLLENSSDGH<br>SERLFGIGFGPYIIA<br>NQHLFRRNNGELTI<br>KTMHGEFKVKNST<br>QLQMKPVEGRDIIVI<br>KMAKDFPPFPQKLK<br>FRQPTIKDRVCMVS<br>TNFQQSGGGSSET<br>VRFQSGSGSISLIAA<br>LAVDYVIGMENAMP<br>WNLPADLAWFKRN<br>TLNKPVIMGRHTWE

			SIGRPLPGRKNIILS<br>SQPSTDDRVTWVK<br>SVDEAIAACGDVPEI<br>MVIGGGRVIEQFLP<br>KAQKLYLTHIDAEVE<br>GDTHFPDYEPDDW<br>ESVFSEFHDADAQN<br>SHSYCFEILERR
N_{22}^{D}	DHD101A-DHD37B-nTEVP-TEVcs-DHFR	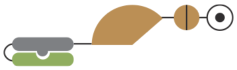	DEKDYHRRLIEHLE<br>DLVRRHEELIKRQK<br>KVVEELERRGLDER<br>LRRVVDRFRRSSER<br>WEEVIERFRQVVDK<br>LRKSVEGSEGSGSE<br>GSGSDDKELDKLLD<br>TLEKILQTATKIIDDA<br>NKLLEKLRRSERKD<br>PKVVETYVELLKRH<br>EKAVKELLEIAKTHA<br>KKVEGSEGSGSEG<br>SGESLFKGPRDYNP<br>ISSTICHLTNESDGH<br>TTSLYGIGFGPFIITN<br>KHLFRRNNGTLLVQ<br>SLHGVFKVKNTTTL<br>QQHLIDGRDMIIIRM<br>PKDFPPFPQKLKFR<br>EPQREERICLVTTNF<br>QTGGGSSENLYFQ<br>SGSGSISLIAALAVD<br>YVIGMENAMPWNL<br>PADLAWFKRNTLNK<br>PVIMGRHTWESIGR<br>PLPGRKNIILSSQPS<br>TDDRVTWVKSVDE<br>AIAACGDVPEIMVIG<br>GGRVIEQFLPKAQK<br>LYLTHIDAEVEGDTH<br>FPDYEPDDWESVF<br>SEFHDADAQNSHS<br>YCFEILERR
N_{11}	DHD15B-DHD37B-nTVMVP	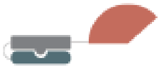	GTERKLLERSRRLQ<br>EESKRLLDEMAEIM<br>RRIKKLLKKARGAD<br>EKVLDELRKIIERIRE<br>LLDRSRKIHERSEEI<br>AYKEEGSEGSGSE<br>GSGSDDKELDKLLD

			TLEKILQTATKIIDDANKLLEKLRRSERKDPKVVETYVELLKRHEKAVKELLEIAKTHAKKVEGSEGSGSEGSSKALLKGVRDFNPISACVCLLENSSDGHSERLFGIGFGPYIIANQHLFRRNNGELTIKTMHGEFKVKNSTQLQMKPVEGRDIIVIKMAKDFPPFPQKLKFRQPTIKDRVCMVSTNFQQS
N_{12}	DHD15B-DHD37B-nTEVP	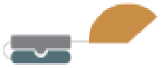	GTERKLLERSRRLQEESKRLLDEMAEIMRRIKKLLKKARGADEKVLDELRKIIERIRELLDRSRKIHERSEEIAYKEEGSEGSGSEGSGSDDKELDKLLDTLEKILQTATKIIDDANKLLEKLRRSERKDPKVVETYVELLKRHEKAVKELLEIAKTHAKKVEGSEGSGSEGSGESLFKGPRDYNPISSTICHLTNESDGHTTSLYGIGFGPFIITNKHLFRRNNGTLLVQSLHGVFKVKNTTTLQQHLIDGRDMIIIRMPKDFPPFPQKLKFREPQREERICLVTTNFQT
C_1	DHD37A-TEVcs-cTVMVP	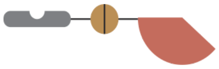	DSDEHLYKLKTFLENLRRHLDRLDKHIKQLRDILSENPEDERVKDVIDLSERSVRIVKTVIKIFEDSVRKKEGSEGSGSEGSENLYFQSGSKSVSSLVSESSHIVHKEDTSFWQHWITTKDGQCGSPLVSIIDGNILGIHSLTHTTNGSNYFVEFPEKFVATYLDAADGWCKNWKFNADKISW

			GSFTLVEDAPEDDF<br>MAKKTVAAIMD
C_2	DHD37A-TVMVcs-cTEVP	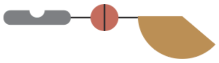	DSDEHLYKLKTFLE<br>NLRRHLDRLDKHIK<br>QLRDILSENPEDER<br>VKDVIDLSERSVRIV<br>KTVIKIFEDSVRKKE<br>GSEGSGSEGSETV<br>RFQSGSKSMSSMV<br>SDTSCTFPSSDGIF<br>WKHWIQTKDGQCG<br>SPLVSTRDGFIVGIH<br>SASNFTNTNNYFTS<br>VPKNFMELLTNQEA<br>QQWVSGWRLNADS<br>VLWGGHKVFMVKP<br>EEPFQPVKEATQLM<br>NS
P_{N1}	nTVMVP	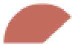	SKALLKGVRDFNPIS<br>ACVCLLENSSDGHS<br>ERLFGIGFGPYIIAN<br>QHLFRRNNGELTIK<br>TMHGEFKVKNSTQL<br>QMKPVEGRDIIVIKM<br>AKDFPPFPQKLKFR<br>QPTIKDRVCMVSTN<br>FQQS
P_{C1}	cTVMVP	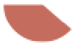	KSVSSLVSESSHIVH<br>KEDTSFWQHWITTK<br>DGQCGSPLVSIIDG<br>NILGIHSLTHTTNGS<br>NYFVEFPEKFVATYL<br>DAADGWCKNWKFN<br>ADKISWGSFTLVED<br>APEDDFMAKKTVAA<br>IMD
P_{N2}	nTEVP	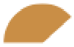	GESLFKGPRDYNPI<br>SSTICHLTNESDGH<br>TTSLYGIGFGPFIITN<br>KHLFRRNNGTLLVQ<br>SLHGVFKVKNTTTL<br>QQHLIDGRDMIIIRM<br>PKDFPPFPQKLKFR<br>EPQREERICLVTTNF<br>QT

P_{C2}	cTEVP	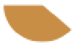	KSMSSMVSDTSCTFPSSDGIFWKHWIQTKDGQCGSPLVSTRDGFIVGIHSASNFTNTNNYFTSVPKNFMELLTNQEAQQWVSGWRLNADSVLWGGHKVFMVKPEEPFQPVKEATQLMN
DP_{N1}	DHD37B-nTVMVP	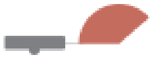	GSDDKELDKLLDTLEKILQTATKIIDDANKLLEKLRRSERKDPKVVETYVELLKRHEKAVKELLEIAKTHAKKVEGSEGSGSEGSSKALLKGVRDFNPISACVCLLENSSDGHSERLFGIGFGPYIIANQHLFRRNNGELTIKTMHGEFKVKNSTQLQMKPVEGRDIIVIKMAKDFPPFPQKLKFRQPTIKDRVCMVSTNFQQS
DP_{C1}	DHD37A-cTVMVP	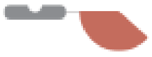	DSDEHLYKLKTFLENLRRHLDRLDKHIKQLRDILSENPEDERVKDVIDLSERSVRIVKTVIKIFEDSVRKKEGSEGSGSEGSKSVSSLVSESSHIVHKEDTSFWQHWITTKDGQCGSPLVSIIDGNILGIHSLTHTTNGSNYFVEFPEKFVATYLDAADGWCKNWKFNADKISWGSFTLVEDAPEDDFMAKKTVAAIMD
DP_{N2}	DHD37B-nTEVP	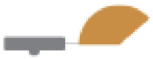	GSDDKELDKLLDTLEKILQTATKIIDDANKLLEKLRRSERKDPKVVETYVELLKRHEKAVKELLEIAKTHAKKVEGSEGSGSEGSGESLFKGPRDYNPISSTICHLTNESDGHTT

			SLYGIGFGPFIITNKH<br>LFRRNNGTLLVQSL<br>HGVFKVKNTTTLQQ<br>HLIDGRDMIIIRMPK<br>DFPPFPQKLKFREP<br>QREERICLVTTNFQ<br>T
DP_{C2}	DHD37A-cTEVP	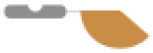	DSDEHLYKLKTFLE<br>NLRRHLDRLDKHIK<br>QLRDILSENPEDER<br>VKDVIDLSERSVRIV<br>KTVIKIFEDSVRKKE<br>GSEGSGSEGSKSM<br>SSMVSDTSCTFPSS<br>DGIFWKHWIQTKDG<br>QCGSPLVSTRDGFI<br>VGIHSASNFTNTNN<br>YFTSVPKNFMELLT<br>NQEAQQWVSGWR<br>LNADSVLWGGHKV<br>FMVKPEEPFQPVKE<br>ATQLMN
T_1	DHD15B-DHD37B-n<br>TVMVP-DHFR	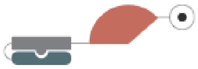	GTERKLLERSRRLQ<br>EESKRLLDEMAEIM<br>RRIKKLLKKARGAD<br>EKVLDELRKIIERIRE<br>LLDRSRKIHERSEEI<br>AYKEEGSEGSGSE<br>GSGSDDKELDKLLD<br>TLEKILQTATKIIDDA<br>NKLLEKLRRSERKD<br>PKVVETYVELLKRH<br>EKAVKELLEIAKTHA<br>KKVEGSEGSGSEG<br>SSKALLKGVRDFNPI<br>SACVCLLENSSDGH<br>SERLFGIGFGPYIIA<br>NQHLFRRNNGELTI<br>KTMHGEFKVKNST<br>QLQMKPVEGRDIIVI<br>KMAKDFPPFPQKLK<br>FRQPTIKDRVCMVS<br>TNFQQSGSGSISLIA<br>ALAVDYVIGMENAM<br>PWNLPADLAWFKR<br>NTLNKPVIMGRHTW<br>ESIGRPLPGRKNIIL<br>SSQPSTDDRVTWV

			KSVDEAIAACGDVP EIMVIGGGRVIEQFL PKAQKLYLTHIDAEV EGDTHFPDYEPDD WESVFSEFHDADA QNSHSYCFEILERR
T_2	DHD15B-DHD37B-nTEVP-DHFR	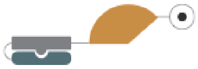	GTERKLLERSRRLQ EESKRLLDEMAEIM RRIKKLLKKARGAD EKVLDELRKIIERIRE LLDRSRKIHERSEEI AYKEEGSEGSGSE GSGSDDKELDKLLD TLEKILQTATKIIDDA NKLLEKLRRSERKD PKVVETYVELLKRH EKAVKELLEIAKTHA KKVEGSEGSGSEG SGESLFKGPRDYNP ISSTICHLTNESDGH TTSLYGIGFGPFIITN KHLFRRNNGTLLVQ SLHGVFKVKNTTTL QQHLIDGRDMIIIRM PKDFPPFPQKLKFR EPQREERICLVTTNF QTGGGSSISLIAALA VDYVIGMENAMPW NLPADLAWFKRNTL NKPVIMGRHTWESI GRPLPGRKNIILSSQ PSTDDRVTWVKSV DEAIAACGDVPEIM VIGGGRVIEQFLPKA QKLYLTHIDAEVEGD THFPDYEPDDWES VFSEFHDADAQNSH SYCFEILERR
N/A	BFP	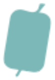	VSKGEELIKENMHM KLYMEGTVDNHHFK CTSEGEGKPYEGT QTMRIKVVEGGPLP FAFDILATSFLYGSK TFINHTQGIPDFFKQ SFPEGFTWERVTTY EDGGVLTATQDTSL QDGCLIYNVKIRGV NFTSNGPVMQKKTL

			GWEAFTETLYPADG<br>GLEGRNDMALKLVG<br>GSHLIANAKTTYRS<br>KKPAKNLKMPGVYY<br>VDYRLERIKEANNE<br>TYVEQHEVAVARYC<br>DLPSKLGHKLN
N/A	mCherry	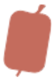	GGVSKGEEDNMAII<br>KEFMRFKVHMEGS<br>VNGHEFEIEGEGEG<br>RPYEGTQTAKLKVT<br>KGGPLPFAWDILSP<br>QFMYGSKAYVKHPA<br>DIPDYLKLSFPEGFK<br>WERVMNFEDGGVV<br>TVTQDSSLQDGEFI<br>YKVKLRGTNFPSDG<br>PVMQKKTMGWEAS<br>SERMYPEDGALKG<br>EIKQRLKLKDGGHY<br>DAEVKTTYKAKKPV<br>QLPGAYNVNIKLDIT<br>SHNEDYTIVEQYER<br>AEGRHSTGGMDEL<br>YKS
N/A	Citrine	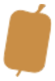	VSKGEELFTGVVPIL<br>VELDGDVNGHKFSV<br>SGEGEGDATYGKLT<br>LKFICTTGKLPVPW<br>PTLVTTFGYGLMCF<br>ARYPDHMKQHDFF<br>KSAMPEGYVQERTI<br>FFKDDGNYKTRAEV<br>KFEGDTLVNRIELKG<br>IDFKEDGNILGHKLE<br>YNYNSHNVYIMADK<br>QKNGIKVNFKIRHNI<br>EDGSVQLADHYQQ<br>NTPIGDGPVLLPDN<br>HYLSYQSALSKDPN<br>EKRDHMVLLEFVTA<br>AGITLGMDELYKS
N/A	miRFP680	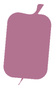	MAEGSVARQPDLLT<br>CDDEPIHIPGAIQPH<br>GLLLALAADMTIVAG<br>SDNLPELTGLAIGAL<br>IGRSAADVFDSETH

			NRLTIALAEPGAAVG<br>APITVGFTMRKDAG<br>FIGSWHRHDQLIFLE<br>LEPPQRDVAEPQAF<br>FRRTNSAIRRLQAA<br>ETLESACAAAAQEV<br>RKITGFDRVMIYRFA<br>SDFSGEVIAEDRCA<br>EVESKLGLHYPAST<br>VPAQARRLYTINPV<br>RIIPDINYRPVPVTP<br>DLNPVTGRPIDLSFA<br>ILRSVSPCHLEFMR<br>NIGMHGTMSISILRG<br>ERLWGLIVCHHRTP<br>YYVDLDGRQACKR<br>VAERLATQIGVMEE
N/A	TVMVcs-Degron-mCherry	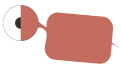	ETVRFQYHKSGAW<br>KLPVSLVKGGTSVS<br>KGEEDNMAIIKEFM<br>RFKVHMEGSVNGH<br>EFEIEGEGEGRPYE<br>GTQTAKLKVTKGGP<br>LPFAWDILSPQFMY<br>GSKAYVKHPADIPD<br>YLKLSFPEGFKWER<br>VMNFEDGGVVTVT<br>QDSSLQDGEFIYKV<br>KLRGTNFPSDGPV<br>MQKKTMGWEASSE<br>RMYPEDGALKGEIK<br>QRLKLKDGGHYDA<br>EVKTTYKAKKPVQL<br>PGAYNVNIKLDITSH<br>NEDYTIVEQYERAE<br>GRHSTGGMDELYK<br>S
N/A	TEVcs-Degron-Citrine	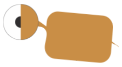	ENLYFQYHKSGAW<br>KLPVSLVKGGGSVS<br>KGEELFTGVVPILVE<br>LDGDVNGHKFSVS<br>GEGEGDATYGKLTL<br>KFICTTGKLPVPWP<br>TLVTTFGYGLMCFA<br>RYPDHMKQHDFFK<br>SAMPEGYVQERTIF<br>FKDDGNYKTRAEVK<br>FEGDTLVNRIELKGI

			DFKEDGNILGHKLE YNYNSHNVYIMADK QKNGIKVNFKIRHNI EDGSVQLADHYQQ NTPIGDGPVLLPDN HYLSYQSALSKDPN EKRDHMVLLEFVTA AGITLGMDELYKS
N/A	HRV3Ccs-Degron-miRFP680	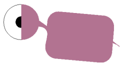	LEVLFQCPHKSGA WKLPVSLVKGGGS MAEGSVARQPDLLT CDDEPIHIPGAIQPH GLLLALAADMTIVAG SDNLPELTGLAIGAL IGRSAADVFDSETH NRLTIALAEPGAAVG APITVGFTMRKDAG FIGSWHRHDQLIFLE LEPPQRDVAEPQAF FRRTNSAIRRLQAA ETLESACAAAAQEV RKITGFDRVMIYRFA SDFSGEVIAEDRCA EVESKLGLHYPAST VPAQARRLYTINPV RIIPDINYRPVPVTP DLNPVTGRPIDLSFA ILRSVSPCHLEFMR NIGMHGTMSISILRG ERLWGLIVCHHRTP YYVDLDGRQACKR VAERLATQIGVMEE
N/A	TEVcs-Casp3	N/A	MGSENTENSVDSK SIKNLEPKIIHGSES MDSGISLDNSYKMD YPEMGLCIIINNKNF HKSTGMTSRSGTD VDAANLRETFRNLK YEVRNKNDLTREEI VELMRDVSKEDHSK RSSFVCVLLSHGEE GIIFGTNGPVDLKKI TNFFRGDRCRSLTG KPKLFIIQACRGTEL DCGIETENLYFQSG VDDDMACHKIPVEA DFLYAYSTAPGYYS WRNSKDGSWFIQS

			LCAMLKQYADKLEF<br>MHILTRVNRKVATEF<br>ESFSFDATFHAKKQI<br>PCIVSMLTKELYFYH
N/A	TEVP	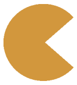	GESLFKGPRDYNPI<br>SSTICHLTNESDGH<br>TTSLYGIGFGPFIITN<br>KHLFRRNNGTLLVQ<br>SLHGVFKVKNTTTL<br>QQHLIDGRDMIIIRM<br>PKDFPPFPQKLKFR<br>EPQREERICLVTTNF<br>QTKSMSSMVSDTS<br>CTFPSSDGIFWKHW<br>IQTKDGQCGSPLVS<br>TRDGFIVGIHSASNF<br>TNTNNYFTSVPKNF<br>MELLTNQEAQQWV<br>SGWRLNADSVLWG<br>GHKVFMVKPEEPF<br>QPVKEATQLMN
N/A	TVMVP	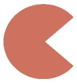	SKALLKGVRDFNPIS<br>ACVCLLENSSDGHS<br>ERLFGIGFGPYIIAN<br>QHLFRRNNGELTIK<br>TMHGEFKVKNSTQL<br>QMKPVEGRDIIVIKM<br>AKDFPPFPQKLKFR<br>QPTIKDRVCMVSTN<br>FQQKSVSSLVSESS<br>HIVHKEDTSFWQH<br>WITTKDGQCGSPLV<br>SIIDGNILGIHSLTHT<br>TNGSNYFVEFPEKF<br>VATYLDAADGWCK<br>NWKFNADKISWGS<br>FTLVEDAPEDDFMA<br>KKTVAAIMD
N/A	HRV3CP	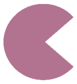	PNTEFALSLLRKNIM<br>TITTSKGEFTGLGIH<br>DRVCVIPTHAQPGD<br>DVLVNGQKIRVKDK<br>YKLVDPENINLELTV<br>LTLDRNEKFRDIRGF<br>ISEDLEGVDATLVVH<br>SNNFTNTILEVGPVT<br>MAGLINLSSTPTNR

			MIRYDYATKTGQCG<br>GVLCATGKIFGIHVG<br>GNGRQGFSAQLKK<br>QYFVEKQ
N_{33}^{D}	nHRV3CP-DHD37B-<br>HRV3Ccs-DHFR	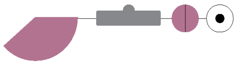	PNTEFALSLLRKNIM<br>TITTSKGEFTGLGIH<br>DRVCVIPTHAQPGD<br>DVLVNGQKIRVKDK<br>YKLVDPENINLELTV<br>LTLDRNEKGSEGSG<br>SEGSGSDDKELDKL<br>LDTLEKILQTATKIID<br>DANKLLEKLRRSER<br>KDPKVVETYVELLK<br>RHEKAVKELLEIAKT<br>HAKKVEGSEGSGS<br>EGSGGGSSLEVLFQ<br>GPGSGSISLIAALAV<br>DYVIGMENAMPWN<br>LPADLAWFKRNTLN<br>KPVIMGRHTWESIG<br>RPLPGRKNIILSSQP<br>STDDRVTWVKSVD<br>EAIAACGDVPEIMVI<br>GGGRVIEQFLPKAQ<br>KLYLTHIDAEVEGDT<br>HFPDYEPDDWESV<br>FSEFHDADAQNSHS<br>YCFEILERR
C_3	DHD37A-TEVcs-TV<br>MVcs-cHRV3CP	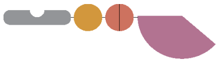	DSDEHLYKLKTFLE<br>NLRRHLDRLDKHIK<br>QLRDILSENPEDER<br>VKDVIDLSERSVRIV<br>KTVIKIFEDSVRKKE<br>GSEGSGSEGSENL<br>YFQSGSETVRFQS<br>GSFRDIRGFISEDLE<br>GVDATLVVHSNNFT<br>NTILEVGPVTMAGLI<br>NLSSTPTNRMIRYD<br>YATKTGQCGGVLCA<br>TGKIFGIHVGGNGR<br>QGFSAQLKKQYFVE<br>KQ
N/A	DHD37A-HRV3Ccs-<br>TEVcs-cTVMVP	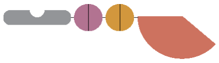	DSDEHLYKLKTFLE<br>NLRRHLDRLDKHIK<br>QLRDILSENPEDER

			VKDVIDLSERSVRIV<br>KTVIKIFEDSVRKKE<br>GSEGSGSEGSLEVL<br>FQGPENLYFQSGSK<br>SVSSLVSESSHIVHK<br>EDTSFWQHWITTKD<br>GQCGSPLVSIIDGNI<br>LGIHSLTHTTNGSNY<br>FVEFPEKFVATYLDA<br>ADGWCKNWKFNAD<br>KISWGSFTLVEDAP<br>EDDFMAKKTVAAIM<br>D
N/A	DHD37A-TVMVcs-H<br>RV3Ccs-cTEVP	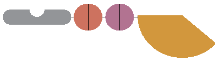	DSDEHLYKLKTFLE<br>NLRRHLDRLDKHIK<br>QLRDILSENPEDER<br>VKDVIDLSERSVRIV<br>KTVIKIFEDSVRKKE<br>GSEGSGSEGSETV<br>RFQSGSLEVLFQGP<br>KSMSSMVSDTSCTF<br>PSSDGIFWKHWIQT<br>KDGQCGSPLVSTR<br>DGFIVGIHSASNFTN<br>TNNYFTSVPKNFME<br>LLTNQEAQQWVSG<br>WRLNADSVLWGGH<br>KVFMVKPEEPFQPV<br>KEATQLMN

Table S3. Plasmids and mRNAs used in transfection experiments.

Figure panel	Plasmids/mRNAs used
3B	1. DP_{N1}, 50 ng; DP_{C1}, 50 ng<br>2. N_{11}^{D}, 50 ng; C_1 50 ng<br>3. N_{11}^{D}, 50 ng; C_1 50 ng; X_1, 400ng<br>4. X_1, 50 ng; T_1, 50 ng; C_1, 50 ng<br>5. X_1, 50 ng; (N_{11}^{D}+ C_1), 50 ng; (N_{12}^{D} + C_2), 450 ng<br>6. DP_{N1}, 50 ng; DP_{C1}, 50 ng
3C	mRNAs<br>X_1, 200 ng; DP_{C1} 200 ng; DP_{C2} 200 ng

<table>
<tr><td></td><td>N_{11} and N_{12} are varied base on the following table:

<table>
<tr><td>N_{11} (ng)</td><td>N_{12} (ng)</td></tr>
<tr><td>500</td><td>0</td></tr>
<tr><td>450</td><td>50</td></tr>
<tr><td>400</td><td>100</td></tr>
<tr><td>300</td><td>200</td></tr>
<tr><td>250</td><td>250</td></tr>
<tr><td>200</td><td>300</td></tr>
<tr><td>100</td><td>400</td></tr>
<tr><td>50</td><td>450</td></tr>
<tr><td>0</td><td>500</td></tr>
</table></td></tr>
<tr><td>3D</td><td>(N_{11}^D+ C_1), 50 ng; (N_{21}^D+ C_1), 3.3 ng;
(N_{12}^D+ C_2), 5 ng; (N_{22}^D+ C_2), 33 ng;

Amount of input proteins are indicated in the figure</td></tr>
<tr><td>3E</td><td>(N_{11}^D+ C_1), 50 ng; (N_{21}^D+ C_1), 50 ng;
(N_{12}^D+ C_2), 16 ng; (N_{22}^D+ C_2), 16 ng;

Amount of input proteins are indicated in the figure</td></tr>
<tr><td>3F</td><td>5:1 -- (N_{11}^D+ C_1), 50 ng; (N_{22}^D+ C_2), 10 ng
2:1 -- (N_{11}^D+ C_1), 50 ng; (N_{22}^D+ C_2), 25 ng
1.5:1 -- (N_{11}^D+ C_1), 50 ng; (N_{22}^D+ C_2), 33 ng
1:1 -- (N_{11}^D+ C_1), 50 ng; (N_{22}^D+ C_2), 50 ng
1:1.5 -- (N_{11}^D+ C_1), 33 ng; (N_{22}^D+ C_2), 50 ng
1:2 -- (N_{11}^D+ C_1), 25 ng; (N_{22}^D+ C_2), 50 ng
1:5 -- (N_{11}^D+ C_1), 10 ng; (N_{22}^D+ C_2), 50 ng

Amount of input proteins are indicated in the figure</td></tr>
<tr><td>3H</td><td>mCherry co-transfection marker: 50 ng
TEVP-activatable Caspase-3: 300 ng</td></tr>
</table>

	Nodes (Y_1 and Y_2): 150 ng each<br><br>Amounts of input proteins (X_1 and X_2) are indicated in the figure.
3I	mCherry co-transfection marker: 50 ng<br>TEVP-activatable Caspase-3: 100 ng<br>Nodes (Y_1 and Y_2): 50 ng each<br><br>Amounts of input proteins (X_1 and X_2) are indicated in the figure.
4C	(N_{11}^D+ C_1), 60 ng; (N_{22}^D+ C_2), 60 ng;<br>(N_{33}^D+ C_3), 60 ng<br><br>Amount of input proteins are indicated in the figure
S4B	1. DP_{N1}, 50 ng; DP_{C1}, 50 ng<br>2. DP_{N2}, 50 ng; DP_{C2}, 50 ng
S4C	1. P_{N2}, 50 ng; P_{C2}, 50 ng<br>2. N_{22}^D, 50 ng; C_2 50 ng<br>3. N_{22}^D, 50 ng; C_2 50 ng; X_2, 400ng<br>4. DP_{N2}, 50 ng; DP_{C2}, 50 ng
S4D	Left:<br>1. P_{N2}, 50 ng; P_{C2}, 50 ng<br>2. N_{12}^D, 50 ng; C_2 50 ng<br>3. N_{12}^D, 50 ng; C_2 50 ng; X_1, 400ng<br>4. DP_{N2}, 50 ng; DP_{C2}, 50 ng<br><br>Middle:<br>5. P_{N1}, 50 ng; P_{C1}, 50 ng<br>6. N_{21}^D, 50 ng; C_1 50 ng<br>7. N_{21}^D, 50 ng; C_1 50 ng; X_2, 400ng<br>8. DP_{N1}, 50 ng; DP_{C1}, 50 ng<br><br>Right:<br>9. P_{N2}, 50 ng; P_{C2}, 50 ng<br>10. N_{22}^D, 50 ng; C_2 50 ng<br>11. N_{22}^D, 50 ng; C_2 50 ng; X_2, 400ng<br>12. DP_{N2}, 50 ng; DP_{C2}, 50 ng
S4E	1. P_{N2}, 50 ng; P_{C2}, 50 ng<br>2. X_1, 50 ng; T_2, 50 ng; C_2, 50 ng<br>3. X_1, 50 ng; (N_{12}^D + C_2), 50 ng; (N_{11}^D+ C_1), 450 ng<br>4. DP_{N2}, 50 ng; DP_{C2}, 50 ng
S4F	$X_1 > X_2$: (N_{11}^D+ C_1), 50 ng; (N_{22}^D+ C_2), 50 ng;

	X_1, 320 ng<br><br>$X_1 = X_2$: (N_{11}^D+ C_1), 50 ng; (N_{22}^D+ C_2), 50 ng; X_1, 320 ng; X_2, 320 ng;<br><br>$X_1 < X_2$: (N_{11}^D+ C_1), 50 ng; (N_{22}^D+ C_2), 50 ng; X_2, 320 ng
S4G	(N_{11}^D+ C_1), 50 ng; (N_{22}^D+ C_2), 50 ng; X_1, 320 ng
S5C	Column 1: Full-length TEVP, 200 ng;<br>Column 2: Full-length TVMVP, 200 ng;<br>Column 3: Full-length HRV3CP: 200 ng;<br><br>BFP co-transfection marker: 10 ng
